# LRF maintains genome integrity by regulating the non-homologous end joining pathway of DNA repair

**DOI:** 10.1038/ncomms9325

**Published:** 2015-10-08

**Authors:** Xue-Song Liu, Gurushankar Chandramouly, Emilie Rass, Yinghua Guan, Guocan Wang, Robin M. Hobbs, Anbazhagan Rajendran, Anyong Xie, Jagesh V. Shah, Anthony J. Davis, Ralph Scully, Andrea Lunardi, Pier Paolo Pandolfi

**Affiliations:** 1 Cancer Research Institute, Beth Israel Deaconess Cancer Center, Department of Medicine and Pathology, Beth Israel Deaconess Medical Center, Harvard Medical School, Boston, Massachusetts 02215, USA; 2 Cancer Research Institute, Beth Israel Deaconess Cancer Center, Department of Medicine, Beth Israel Deaconess Medical Centre, Harvard Medical School, Boston, Massachusetts 02215, USA; 3 Department of Systems Biology, Harvard Medical School, 4 Blackfan Circle, HIM 564, Boston, MA 02115, USA; 4 Division of Molecular Radiation Biology, Department of Radiation Oncology, University of Texas Southwestern Medical Centre, 2201 Inwood Rd, Dallas, Texas 75390, USA

## Abstract

Leukemia/lymphoma-related factor (LRF) is a POZ/BTB and Krüppel (POK) transcriptional repressor characterized by context-dependent key roles in cell fate decision and tumorigenesis. Here we demonstrate an unexpected transcription-independent function for LRF in the classical non-homologous end joining (cNHEJ) pathway of double-strand break (DSB) repair. We find that LRF loss in cell lines and mouse tissues results in defective cNHEJ, genomic instability and hypersensitivity to ionizing radiation. Mechanistically, we show that LRF binds and stabilizes DNA-PKcs on DSBs, in turn favouring DNA-PK activity. Importantly, LRF loss restores ionizing radiation sensitivity to p53 null cells, making LRF an attractive biomarker to direct p53-null LRF-deficient tumours towards therapeutic treatments based on genotoxic agents or PARP inhibitors following a synthetic lethal strategy.

## Abstract (web summary)

Leukemia/lymphoma-related factor (LRF), a transcriptional repressor, plays key roles in cell fate decision and tumorigenesis. Here, Liu *et al*. show that LRF loss results in defective classical non-homologous end joining, genomic instability and hypersensitivity to ionizing radiation, revealing a transcription-independent regulation of DNA-PK complex.

## Graphical abstract

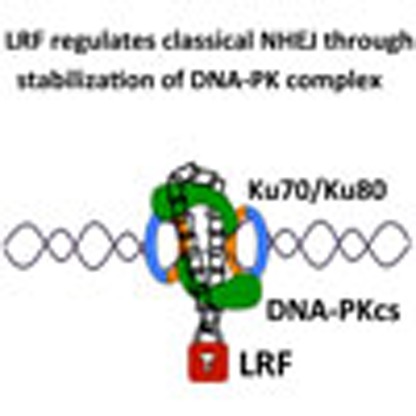

The ability to maintain a stable genome is crucial for normal cell function, and genomic instability may underlie many developmental disorders and human diseases, including cancer^1^. DNA double-strand breaks (DSBs) are perhaps the most deleterious threat to genomic stability. Cells use two main pathways to repair DSBs: non-homologous end joining (NHEJ) and homologous recombination (HR)^2^. These two pathways are largely distinct from one another. HR is particularly effective in S and G2 phases when the break is repaired using genetic information from a sister chromatid, whereas NHEJ can be effective at all times in the cell cycle, yet it is often error prone^3^. The DNA-dependent protein kinase (DNA-PK) complex, including catalytic subunit DNA-PKcs and DNA-binding subunits Ku70/80, is a key component of the classical non-homologous end joining (cNHEJ) apparatus. The physical interaction between DNA-bound Ku (Ku70/Ku80), in particular the C-terminal tail of Ku80, and DNA-PKcs at sites of DNA breaks defines a functional DNA-PK complex that concomitantly bridges the broken DNA ends and activates the DNA repair machinery through the phosphorylation of specific downstream targets^4^,^5^.

LRF (formerly known as POKEMON^6^, FBI-1 (ref. 7) or OCZF^8^) is encoded by the *ZBTB7A* gene, and is a member of the POZ/BTB and Krüppel (POK) family of transcription factors. POK transcription factors can bind DNA through a Krüppel-like-DNA-binding domain and repress transcription by recruiting co-repressor complexes through the POZ (Pox virus and Zinc finger) domain^9^. POK transcription factors have been recognized as critical developmental regulators and have been directly implicated in human cancer^10^. For example, BCL6 (B-Cell Lymphoma 6) and PLZF (Promyelocytic Leukemia Zinc Finger) are critical players in the pathogenesis of Non-Hodgkin's Lymphoma and acute promyelocytic leukemia, respectively^11^,^12^. LRF shares structural similarities with BCL6 and PLZF and plays critical context-dependent role in embryonic development, haematopoiesis and tumorigenesis^6^,^13^,^14^,^15^,^16^,^17^,^18^,^19^.

In this work, we identify a novel and transcriptional independent function for LRF in the maintenance of genomic stability by regulation of cNHEJ. Mechanistically, we demonstrate that LRF is rapidly recruited on the sites of DNA damage where, by binding DNA-PKcs, it stabilizes the DNA-PK complex, in turn promoting DNA-PKcs kinase activity and efficient DSB repair. Importantly, LRF downregulation, a frequent hallmark of different types of human cancer, restores radiation sensitivity in p53 null cells, thus becoming a new potential biomarker of remarkable therapeutic relevance.

## Results

### LRF is required for maintenance of genomic integrity

**Figure 1:**
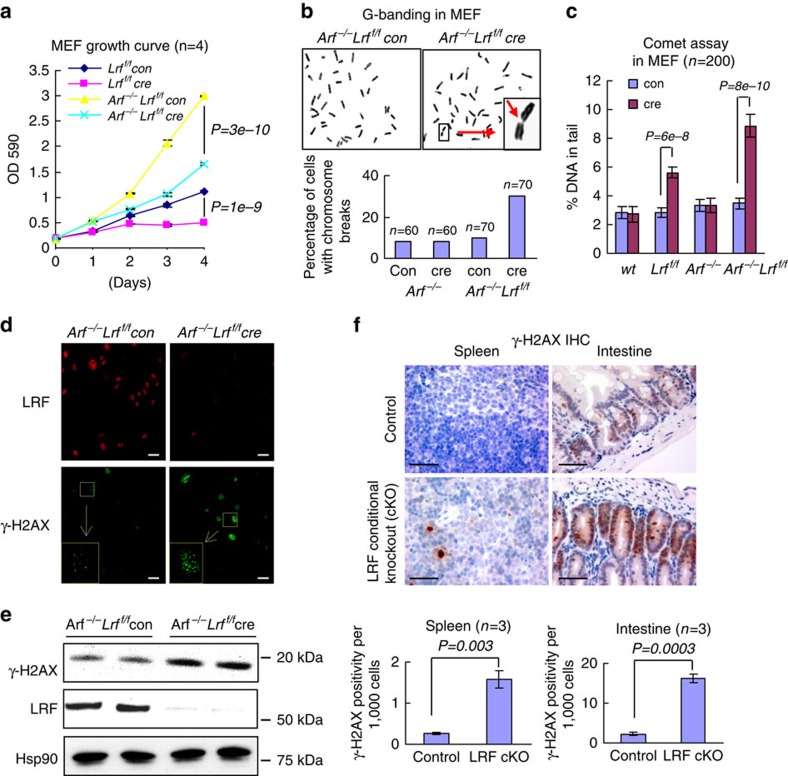
LRF is required for maintenance of genome integrity. (**a**) Growth curve of control and *Lrf* deleted MEFs in both wild-type and *Arf* null backgrounds. Data from *n*=4 independent experiments are presented as mean±s.e.m. Associated *P* value calculated by Student's *t*-test analysis is indicated. (**b**) Metaphase chromosome spreads stained with Giemsa. Insert is an enlargement of a typical chromosome break. Ratio of cells with chromosome breaks was counted in *Arf*^*−/−*^ and *Arf*^*−/−*^ *Lrf*^f/f^ MEFs transduced with Control or Cre expression retrovirus. (**c**) Detection of DSBs by neutral comet assay, in wild-type, *Lrf*^*f/f*^, *Arf*^*−/−*^ and *Arf*^*−/−*^*Lrf*^*f/f*^ MEFs transduced with control or Cre expression retrovirus. The percentage of DNA in comet tails is scored from 200 cells of 3 different experiments and presented as mean±s.e.m. Associated *P* value calculated by Student's *t*-test analysis is indicated. (**d**,**e**) Increased γ-H2AX in *Arf*^*−/−*^ *Lrf* deleted MEFs as shown by immunofluorescence (scale bar, 10 μm) (**d**) and immunoblot (**e**). Hsp90 is used as a loading control for the immunoblot. (**f**) Paraffin sections of *Lrf*^*+/+*^ *Villin-Cre* (control) or *Lrf*^*f/f*^ *Villin-Cre* C57/BL6 mouse intestine (3 months old), *Lrf*^*+/+*^ *Mx1-Cre* (control) or *Lrf*^*f/f*^ *Mx1-Cre* C57/BL6 mouse spleen (4 weeks after four PIPC injection) were immunostained with γ-H2AX antibody (scale bar, 100 μm). The fractions of γ-H2AX positive cells in control and Lrf conditional knockout spleen and intestine were scored. Data from *n*=3 different mice are presented as mean±s.e.m. Associated *P* value calculated by Student's *t*-test analysis is indicated. Scale bar, 100 μm.

LRF is a critical repressor of the tumour suppressor gene *Arf*, and cells such as mouse embryonic fibroblasts (MEFs), which lack Lrf become refractory to oncogenic transformation and undergo premature senescence^6^. In an effort to identify new functions of LRF unrelated to Arf regulation through a clean genetic approach, we compared the effects of acute *Lrf* deletion in *Lrf*^*flox/flox*^ or *Arf*^*−/−*^ *Lrf*^*flox/flox*^ MEFs through infection with a Cre recombinase-containing retrovirus. Although Cre expression in both wild-type and *Arf*^*−/−*^ MEFs had no effect on cell proliferation (Supplementary Fig. 1a), and Cre-mediated deletion of *Lrf* in *Lrf*^*flox/flox*^ MEFs triggered the expected growth suppression through Arf-dependent cellular senescence^6^ (Fig. 1a), surprisingly, loss of Lrf caused a profound growth suppression in the *Arf*^*−/−*^ MEFs as well (Fig. 1a). The growth defect of *Arf*^*−/−*^ *Lrf* deleted (*Arf*^*−/−*^ *Lrf*^*f/f*^ cre) MEFs was accompanied by evidence of chromosome breakage, as shown by Giemsa staining of metaphase chromosome spreads (Fig. 1b). Telomere Fish fluorescent *in situ* hybridization staining of chromosome spreads also indicated accumulation of chromosome breaks, aneuploidy, polyploidy and abnormal chromosomes in *Arf*^*−/−*^ *Lrf* deleted MEFs (Supplementary Fig. 1b). Accordingly, neutral comet assay showed a significant accumulation of DNA DSBs in *Lrf* deleted MEFs (Fig. 1c), and immunofluorescence and western blot studies confirmed a marked increase in γ-H2AX staining (Fig. 1d,e). To further characterize this phenotype, we assessed whether LRF conditional inactivation triggers unrepaired DNA damage *in vivo. Villin-Cre* and *Mx1-Cre* transgenes were used to delete floxed *Lrf* in the mouse intestine and hematopoietic systems, respectively^20^,^21^. Importantly, in LRF conditional knockout intestine and spleen the downregulation of LRF (Supplementary Fig. 1c) was associated with a significant increase of γ-H2AX levels (Fig. 1f), suggestive of persistent DNA damage in these cells^22^.

### LRF deficiency sensitizes cells to ionizing radiation

**Figure 2:**
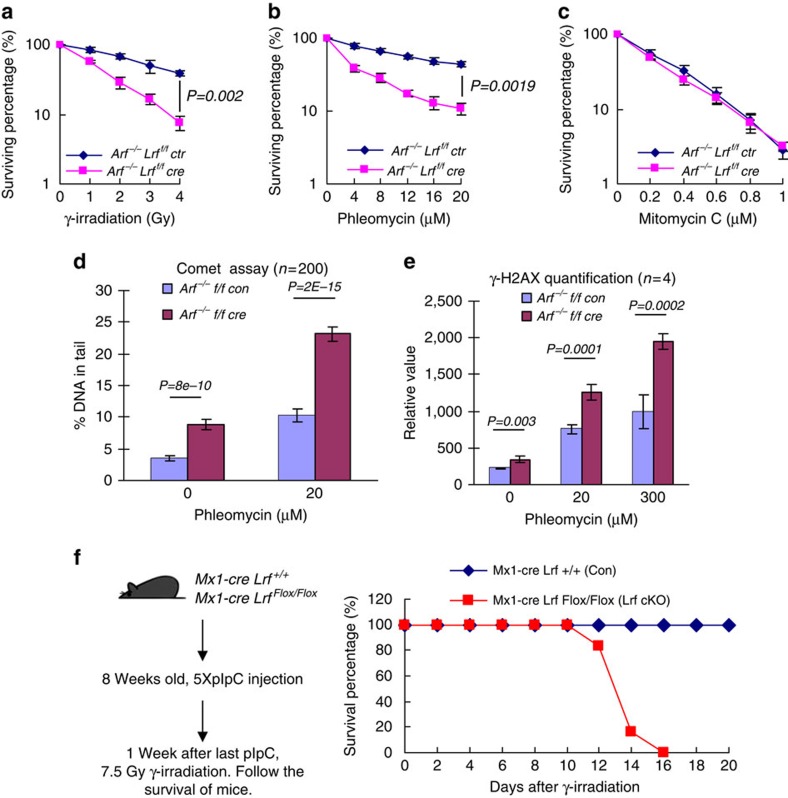
LRF-deficient cells are hypersensitive to ionizing radiation. (**a**–**c**) Clonogenic survival of *Arf*^*−/−*^ and *Arf*^*−/−*^ LRF-deleted MEFs treated with γ-radiation (**a**), phleomycin (**b**) and mitomycin C (**c**). Data from *n*=4 independent experiments are presented as mean±s.e.m. Associated *P* value calculated by Student's *t*-test analysis is indicated. (**d**) DNA damage levels in *Arf*^*−/−*^ and *Arf*^*−/−*^ LRF-deleted MEF treated with phleomycin assessed by comet assay. The percentage of DNA in comet tails is scored from 200 cells of three different experiments and presented as mean±s.e.m. Associated *P* value calculated by Student's *t*-test analysis is indicated. (**e**) γ-H2AX levels assessed by flow cytometric analysis of *Arf*^*−/−*^ and *Arf*^*−/−*^ LRF deleted MEFs, 1 h after 20 or 300 μM phleomycin treatment. Data from *n*=4 independent experiments are presented as mean±s.e.m. Associated *P* value calculated by Student's *t*-test analysis is indicated. (**f**) Survival curve of *Lrf* hematopoietic system conditional knockout mice (*Lrf cKO*) (*n*=6) and sibling control mice (*n*=9) after single dose of whole-body γ-radiation (7.5 Gy).

Since LRF inactivation results in persistent DNA damage and genomic instability, we used clonogenic survival assays to assess the sensitivity of *Arf*^*−/−*^ and *Arf*^*−/−*^ *Lrf* deleted MEFs to different types of DNA-damaging agents. These included γ-radiation, the radiomimetic drug phleomycin, the Topoisomerase II inhibitor ICRF-193, the Topoisomerase I inhibitor Camptothecin, and the DNA cross-linking agent, mitomycin C. Compared with *Arf*^*−/−*^ control MEFs, *Arf*^*−/−*^ *Lrf* deleted cells revealed hypersensitivity to γ-radiation, phleomycin and ICRF-193 (Fig. 2a,b and Supplementary Fig. 2a), but no alteration in mitomycin C and Camptothecin sensitivity (Fig. 2c, and Supplementary Fig. 2b). Furthermore, upon treatment with phleomycin at various concentrations, *Arf*^*−/−*^ *Lrf* deleted MEFs displayed a significant increase of comet tail DNA content and γ-H2AX levels (Fig. 2d,e). We then further tested *in vivo* whether *Lrf* null mutants are hypersensitive to ionizing radiation (IR). Constitutive *Lrf* inactivation results in embryonic lethality^6^, while conditional *Lrf* inactivation in the adult hematopoietic system (‘*Lrf* cKO'), using Mx1-Cre and pIpC induction, is compatible with a normal lifespan^14^. After a single dose of whole-body γ-irradiation (7.5 Gy), all *Lrf cKO* mice died within 16 days, while all wild-type control mice remained healthy for 2 weeks after irradiation (Fig. 2f). After irradiation, *Lrf cKO* bone marrow cells accumulated much more unrepaired DNA damage (shown by γ-H2AX staining) and became apoptotic (by cleaved caspase 3 staining). *Lrf cKO* mice were found to have died from acute bone marrow failure (Supplementary Fig. 2c).

### LRF participates in *Xrcc4*-dependent NHEJ

**Figure 3:**
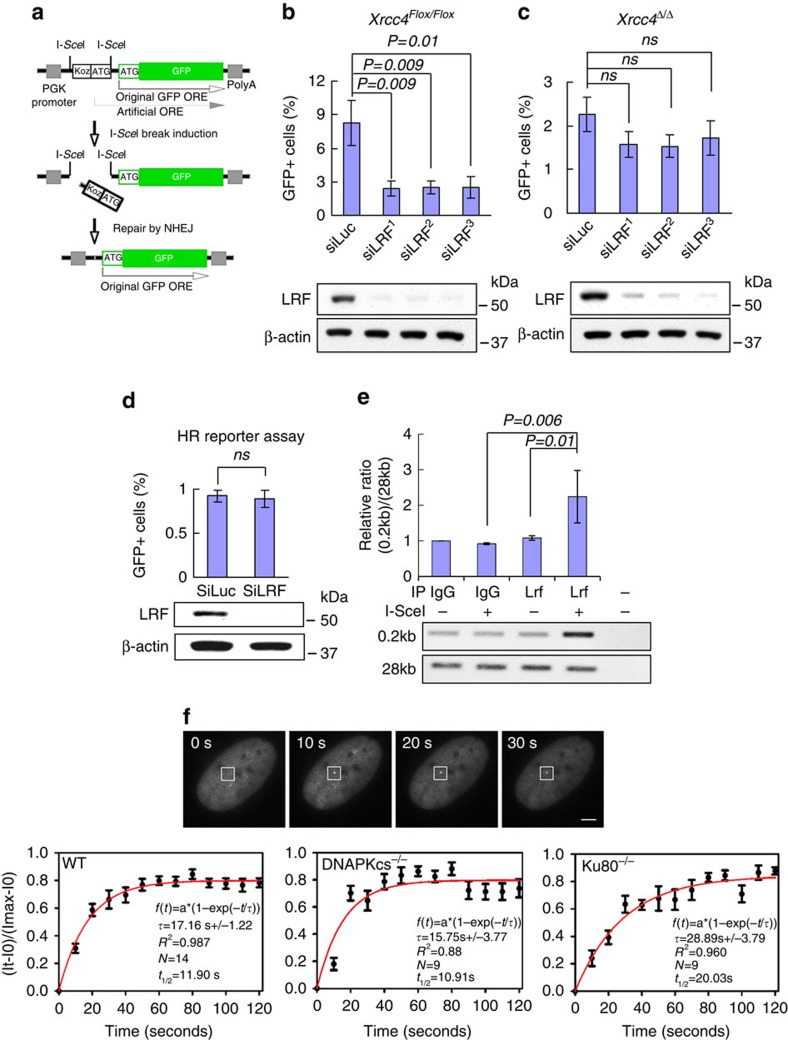
Transcriptional independent role of LRF in cNHEJ. (**a**) Structure of the NHEJ reporter. ‘Koz-ATG' denotes an artificial Kozak-ATG translation start site. ORF (open reading frame); PGK (phosphoglycerate kinase); PolyA (polyadenylation signal). (**b**) *Xrcc4*^Flox/Flox^ ES cells containing a stably integrated NHEJ reporter were subject to LRF knockdown using three independent siRNAs. Statistically significant reductions in GFP-positive cells are shown for each LRF siRNA. Data from four independent experiments are presented as mean values±s.e.m. Associated *P* value calculated by Student's *t*-test analysis is indicated. (**c**) Effect of LRF siRNAs on NHEJ in isogenic *Xrcc4*^Δ/Δ^ NHEJ reporter ES cells. Differences in the amount of GFP-positive siLuc and siLRF transfected cells are not statistically significant. Data from four independent experiments are presented as mean±s.e.m. Associated *P* value calculated by Student's *t*-test analysis is indicated. (**d**) Effect of LRF siRNA on homologous recombination efficiency using a specific reporter assay (see also Supplementary Fig. 3c). Differences in the amount of GFP-positive siLuc and siLRF transfected cells are not statistically significant. Data from 4 independent experiments are presented as mean values±s.e.m. Associated *P* value calculated by Student's *t*-test analysis is indicated. (**e**) Recruitment of Lrf to site-specific DNA breaks generated by I-SceI. ChIP analysis indicates the significant recruitment of LRF to DNA breaks induced by I-SceI. Average values of *n*=3 independent experiments are shown as mean values±s.d. Associated *P* value calculated by Student's *t*-test analysis is indicated. (**f**) GFP-LRF recruitment to DSB sites generated by a multiphoton laser system. LRF kinetics of recruitment to DSBs were evaluated in wild-type, DNA-PKcs^−/−^ and Ku80^−/−^ cells. Average values of *n*=20 independent acquisitions are shown as mean values±s.d. Scale bar, 1 μm

To directly test whether a specific DSB repair process requires LRF function, we next took advantage of selective DSB repair reporter assays (Fig. 3a, and Supplementary Fig. 3c)^23^. Notably, siRNA-mediated knockdown of LRF caused a significant decrease in NHEJ repair efficiency as shown by decreased I-SceI-induced GFP expression in NHEJ reporter cells (Fig. 3b). Classical NHEJ (cNHEJ) is a rapid and efficient process, requiring DNA Ligase IV and XRCC4. In cells lacking either of these genes, rejoining of DSBs occurs through a slower, highly error-prone process termed ‘alternative end joining' (aEJ)^4^,^5^,^24^,^25^,^26^. Using biallelically deleted *Xrcc4* (here termed *Xrcc4*^Δ/Δ^) NHEJ reporter ES cells, we observed only a modest and statistically insignificant decrease of NHEJ efficiency after Lrf knockdown (Fig. 3c). Similarly, pharmacological inhibition of DNA-PKcs activity (NU7441) combined with siRNA-mediated knockdown of LRF showed only a mild and statistically insignificant reduction of NHEJ efficiency in *Xrcc4* proficient ES cells when compared with DNA-PKcs inhibition alone (Supplementary Fig. 3a)^23^. On the other hand, depletion of LRF had no impact on rejoining of I-SceI-induced DSBs in human U2OS cells carrying a reporter of micro-homology-mediated end joining^27^ (Supplementary Fig. 3b), a frequent mediator of aEJ^27^,^28^. We found that LRF is also dispensable for HR, based on experiments performed using mouse ES and U2OS cells carrying an HR reporter^29^ (Fig. 3d and Supplementary Fig. 3c,d).

Collectively, these results clearly define LRF as a novel important player in Xrcc4/DNA-PK-dependent cNHEJ pathway of DSB repair. In future experiments, it will be instructive to define more fully the structure-function analysis of LRF-DNA-PKcs interaction as well as the epistatic relationships between LRF inactivation and the loss of other c-NHEJ genes.

LRF is known to act as a transcription factor. We therefore used microarray analysis in wild-type and *Lrf* conditional knockout MEFs to decrypt its activity in DSB repair (Supplementary Tables 1 and 2). Surprisingly, *Lrf* knockout cells did not display significant alterations in the expression of genes known to be essential for cNHEJ either at early (Supplementary Table 2) or at late passages (Supplementary Fig 3e,f). Only the expression of MRE11, implicated in both aEJ and cNHEJ, resulted downregulated in *Lrf* null cells compared with wild-type (Supplementary Tables 1 and 2). Although MRE11 mild downregulation could explain the slight reduction in the efficiency of aEJ noted in Fig. 3c, the much more pronounced impact on the cNHEJ pathway in LRF-depleted cells (Fig. 3b) suggests a more fundamental role of LRF in this mechanism of DSB repair. Strongly supporting this hypothesis, chromatin immunoprecipitation (ChIP) experiments demonstrated the binding of LRF to site-specific DSBs generated by I-SceI (Fig. 3e), while an *in vivo* imaging approach proved the ability of LRF to localize to the vicinity of DSBs generated by laser damage (Fig. 3f, upper panel), with a kinetic closely comparable to other cNHEJ proteins, such that of Ku80 and DNA-PKcs^30^. Importantly, LRF recruitment to DSBs is not dependent on DNA-PKcs or Ku80 (Fig. 3f, lower panels-middle/right).

Taken together, these results point to a transcriptional independent role for LRF in cNHEJ, a conclusion consistent with the observation that *Lrf*-deleted cells are hypersensitive to IR, phleomycin and ICRF-193, but not to mitomycin C and camptothecin.

### LRF interacts with DNA-PKcs and regulates DNA-PK function

**Figure 4:**
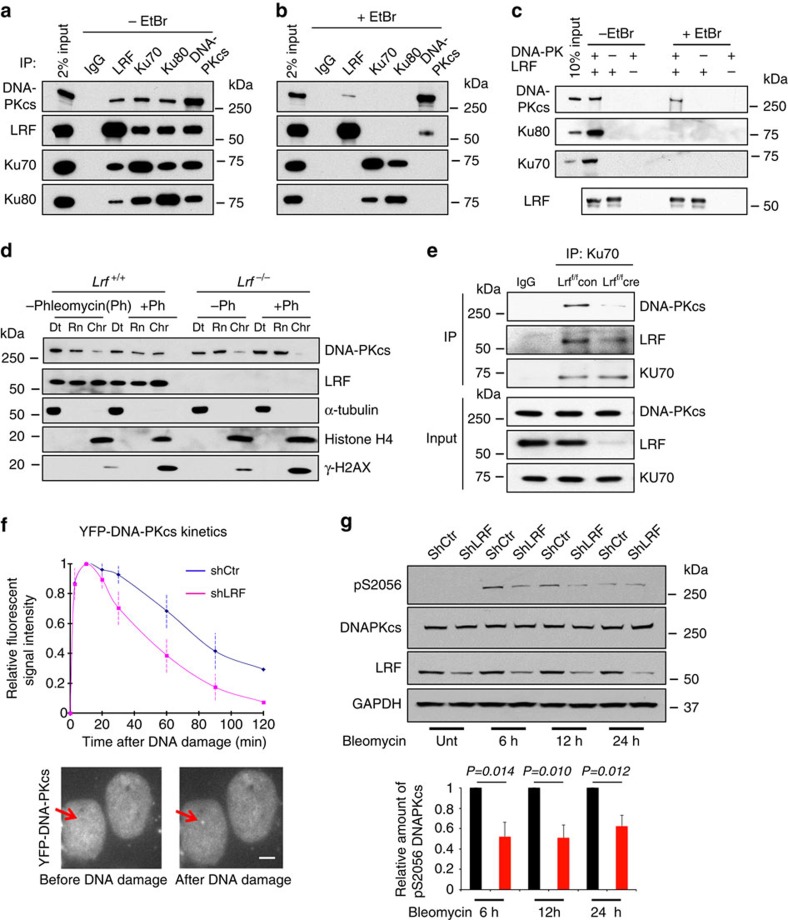
Interaction of LRF with DNA-PKcs and Ku70/80. (**a**,**b**) Endogenous interaction between LRF and DNA-PKcs/Ku70/Ku80 in the absence (**a**) or presence (**b**) of ethidium bromide (EtBr) (50 μg ml^−1^). (**c**) *In vitro* LRF and DNA-PK binding assay. FLAG-tagged LRF was purified by immunoprecipitation with Flag antibody affinity resin then washed with high salt buffer (500 mM NaCl). Purified DNA-PK components were added in the absence or presence of EtBr and the association of Ku70, Ku80 or DNA-PKcs with LRF was assessed by western blotting. (**d**) LRF stabilizes DNA-PKcs on chromatin in response to DNA damage. *p53*^*−/−*^ *Lrf*^*+/+*^ and *p53*^*−/−*^ *Lrf*^*−/−*^ cells were fractionated into detergent extractable (Dt), RNase extractable (Rn) and RNase-resistant chromatin (Chr) compartments. (**e**) Ku70 co-immunoprecipitation performed in control (*p53*^*−/−*^ *Lrf*^*f/f*^ ctr) and LRF conditional knockout (*p53*^*−/−*^ *Lrf*^*f/f*^ cre) MEF. (**f**) YFP tagged DNA-PKcs was expressed in stable sh*Ctr* and sh*LRF* U2OS cells. Association and dissociation kinetics of YFP-DNA-PKcs recruitment to DNA damage foci are shown. Average values of 20 cells are presented as mean values±s.d. Scale bar, 1 μm. (**g**) Relative amount of phosphoSerine2056-DNA-PKcs in stable shCtr and shLRF U2OS cells treated with Bleomycin for 6, 12 and 24 h is shown. Average values of *n*=3 independent experiments are presented as mean values±s.d. Associated *P* value calculated by Student's *t*-test analysis is indicated.

**Figure 5:**
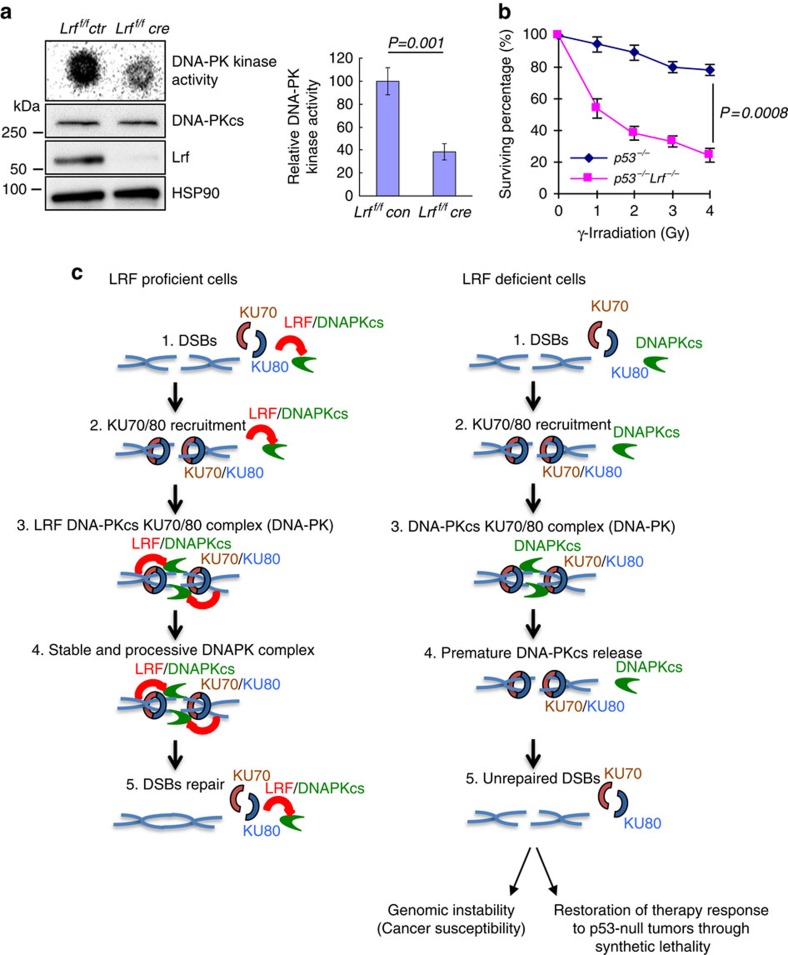
LRF stabilizes the Ku/DNA-PKcs complex on DNA breaks. (**a**) DNA-PK kinase activity was assessed in total cell extracts from *Arf*^*−/−*^ control (*Arf*^*−/−*^ *Lrf*^*f/f*^ con) and *Arf*^*−/−*^ LRF-deleted MEF (*Arf*^*−/−*^ *Lrf*^*f/f*^ Cre). (**b**) Clonogenic survival of *p53*^*−/−*^ *Lrf*^*+/+*^ and *p53*^*−/−*^ *Lrf*^*−/−*^ MEF after different doses of γ-radiation. (**c**) Schematic representation of LRF function in NHEJ DSBs repair pathway. Data are presented as mean±s.e.m. of three independent experiments. Associated *P* value calculated by Student's *t*-test analysis is indicated.

To determine the transcriptional independent role of LRF in cNHEJ, we first assessed whether LRF could associate with DSB repair protein complexes. To this end, LRF-associated proteins were isolated through tandem affinity purification from HeLa cells stably expressing human LRF tagged with Flag and haemagglutinin (HA) epitopes and analysed by mass spectrometry. Importantly, we found LRF associated with the DNA-PK protein complex, including DNA-PKcs, Ku70 and Ku80 (Supplementary Fig. 4a). Mass spectrometry data were then validated in pull-down experiments with overexpressed FLAG/HA tagged LRF (Supplementary Fig. 4b, and Supplementary Table 3), as well as through the reciprocal co-immunoprecipitation of endogenous LRF with DNA-PKcs, Ku70 or Ku80 (Fig. 4a). The association between DNA-PKcs and the Ku70/Ku80 heterodimer is DNA dependent^31^. We therefore determined whether the association between LRF and DNA-PKcs or Ku requires DNA. Endogenous co-immunoprecipitations in the presence of ethidium bromide (50 μg ml^−1^), which disrupts DNA-dependent interactions, indicated that the association between LRF and Ku is strictly dependent on the presence of DNA, while the interaction between LRF and DNA-PKcs, although favoured by DNA, persists in its absence (Fig. 4b). Furthermore, *in vitro* binding with FLAG-tagged LRF and purified DNA-PK components also indicated that the binding of LRF to Ku70 and Ku80 requires DNA, while DNA bridging is not necessary for the interaction between DNA-PKcs and LRF (Fig. 4c). Interestingly, we observed that, in the absence of *Lrf*, the mobilization of DNA-PKcs to the chromatin fraction following DNA damage, is significantly decreased (Fig. 4d, and Supplementary Fig. 4c). Furthermore, significantly less DNA-PKcs was co-immunoprecipitated with Ku antibodies in LRF-depleted cells compared with controls (Fig. 4e and Supplementary Fig. 4d,e). Defects in the formation and stabilization of the Ku/DNA-PKcs/DNA complex may predict impaired DNA-PKcs kinetics in response to DNA damage. To test this hypothesis, we expressed YFP-tagged DNA-PKcs or Ku80 constructs in control and sh*LRF* stable U2OS cells. Strikingly, in a context where the kinetics of recruitment/release of Ku80 were not affected by LRF (Supplementary Fig. 4f), we observed that the retention time, but not the recruitment time, of DNA-PKcs on the laser-induced breaks was significantly decreased in LRF knockdown compared with control cells (Fig. 4f). Endogenous DNA-PKcs autophosphorylation on serine 2056, a known correlate of DNA-PK activity^32^,^33^,^34^,^35^,^36^, was significantly reduced in LRF knockdown compared to control cells following treatment of cells with bleomycin (Fig. 4g). In keeping with these findings, in an *in vitro* assay using purified DNA-PKcs protein and extracts from wild-type and *Lrf*-deleted MEFs, we observed a substantially lower DNA-PKcs kinase activity in the absence of Lrf compared with controls (Fig. 5a).

### LRF loss restores IR sensitivity in *p53* null cells

A characteristic feature of *p53* null cells is their resistance to IR^37^. This effect is reported to require normal DNA-PK function and loss of DNA-PKcs, Ku70 or Ku80 can restore the radiation sensitivity of *p53* null cells^38^. We therefore tested whether LRF loss, which is observed in advanced cancers^15^,^17^,^18^, could restore IR sensitivity in *p53*-deficient cells. Indeed, *Lrf* loss restored IR-induced apoptosis of *p53* null MEFs (Fig. 5b and Supplementary Fig. 4g).

## Discussion

The human genome encodes ∼60 POK family proteins^16^,^19^,^39^, containing an amino-terminal POZ domain and several carboxy-terminal C2H2 Zinc finger domains. POK family proteins have been implicated in embryogenesis, the pathogenesis of cancer and other diseases primarily as transcriptional regulators of gene expression, although ZBTB1 has been recently shown to exert transcription-independent functions intriguingly associated with DNA repair^40^. Even though originally characterized as a proto-oncogene^6^, human *ZBTB7A* is located at 19p13.3, a chromosomal region that is frequently lost in different types of human cancer, including prostate cancer^18^,^41^,^42^. Interestingly, LRF has been recently characterized as a potent context-dependent tumour suppressor through the transcriptional repression of oncogenic pathways and glycolytic metabolism^15^,^16^,^17^,^18^. Here we identify LRF, a bonafide member of the POK family of proteins, as an important regulator of the DNA-PK complex required for the maintenance of genome integrity, which is a novel and unexpected function that LRF exerts independently of its transcriptional function. DNA-PKcs is the largest known protein kinase in the cell, which belongs to the phosphatidylinositol-3 (PI-3) kinase-related-kinase (PIKK) super-family based on primary structure. In current models, Ku association with DNA ends initiates a complex DNA-PKcs-dependent signalling pathway through phosphorylation of downstream effectors responsible for DSB repair^43^. Importantly, this study unravels a novel and unexpected transcriptional independent function of POK family of proteins into the critical cellular processes of DNA-PK function and cNHEJ. Notably, BCL6 (B-cell lymphoma-6), a further member of the POK family and a key oncogenic driver in B-cell lymphoma^44^, has been demonstrated to physically bind LRF^45^ thus suggesting a possible role of BCL6 in the regulation of NHEJ. Importantly, LRF downregulation, caused by genetic loss or other mechanisms, has been recently reported to characterize specific subgroups of cancer patients^15^,^17^,^18^,^46^,^47^. As a novel component of the DNA-PK complex and regulator of DNA-PK stability and activity (Fig. 5c), LRF represents an attractive biomarker with important therapeutic implications since its downregulation might serve to identify those tumours that are particularly dependent on NHEJ activity, such as for instance a subset of *p53*-null cancers, towards therapeutic treatments based on genotoxic agents, radiation, or PARP inhibitors following the synthetic lethality paradigm.

## Methods

### Antibodies and reagents

Anti-Ku70 antibody (1 μg, BD Biosciences #611892), Anti-Ku80 antibody (1 μg, Cell Signaling Technology #2753S), anti-DNA-PKcs antibody (1 μg, Bethyl Laboratories #A300-517A), and anti-LRF antibody (1 μg, Bethyl Laboratories #A300-548A) were used for immunoprecipitation. Anti-Ku70 antibody (1:3,000, Cell Signaling D10A7 #4588), Anti-Ku80 antibody (1:3,000, Cell Signaling Technology #2753S), anti-DNA-PKcs antibody (1:3,000, Santa Cruz G4 #sc-5282), and Anti-LRF antibody (1:1,000 13E9, Pandolfi lab), and anti phosphoS2056-DNA-PKcs (Cell Signaling Technology #4215S), β-actin (1:5,000, Abcam #ab8226), Rad51 (Scully lab; 1:1,000), Brca1 (Scully lab; 1:1,000), LRF (13E9, Pandolfi lab; 1:1,000), Mre11 (Novus #NB100-142; 1:1,000), Xrcc4 (Santa Cruz #sc-8285; 1:1,000), anti-γ-H2AX antibody (1:5,000 Cell Signaling, 20E3) were used for immunoblot. Anti-γ-H2AX antibody (1:50 Cell Signaling, 20E3), anti-LRF antibody (1:50, 13E9 Pandolfi lab), anti-cleaved caspase-3 antibody (1:50, Cell Signaling, 5A1E) were used for immunohistochemistry. Bleomycin, Phleomycin, Mitomycin C, ICRF-193, and Camptothecin were purchased from Sigma. Purified DNA-PK complex was purchased from Promega.

### Retrovirus transduction of mouse embryonic fibroblast

All animal procedures have been approved by the Beth Israel Deaconess Medical Center and Harvard Medical School institutional review board. *Lrf*^*+/−*^, *Lrf*^*flox/flox*^, *Arf*^*−/−*^ and *p53*^*−/−*^ mice are previously described^6^,^14^,^48^,^49^. *p53*^*−/−*^ and *p53*^*−/−*^ *Lrf*^*−/−*^ MEF were prepared from E13.5 mouse embryos obtained from the intercross of *p53*^*−/−*^ *Lrf*^*+/−*^ mice. To generate primary *Lrf*^*flox/flox*^ and *Arf*^*−/−*^ *Lrf*^*flox/flox*^ MEF, *Arf*^*+/−*^ *Lrf*^*flox/flox*^ mice were intercrossed. MEFs were transduced with MSCV-PIG-Cre or empty control vector retrovirus for 2 days at passage 2, and then selected with 2 μg ml^−1^ puromycin for 2 days before use in subsequent experiments.

### Microarray analysis

*Lrf*^*flox/flox*^ MEFs were transduced with MSCV-PIG-Cre or empty control vector retroviruses for 2 days at passage 2. After selection with puromycin for 2 days, total RNAs were purified using the RNAeasy Mini Kit (Qiagen) and treated with RNase-free DNase set (Qiagen). RNAs from two independent experiments were labelled and hybridized using Affymetrix GeneChip HT Mouse Genome 430 arrays by the Beth Israel Deaconess Medical Center Genomics and Proteomics Center. Genes with normalized data values differing by a factor greater or less than 1.5-fold were selected and further evaluated statistically.

### Cell growth assay

Cells were seeded in 12-well plates at a density of 10^4^/well, then left to grow for 4 days. Cells were fixed by paraformaldehyde at each time point, and the cell number determined by crystal violet staining as described^50^.

### Comet assay

DNA lesions were assessed using a single-cell gel electrophoretic comet assay kit (Trevigen). Cells were combined with low melting point agarose and pipetted onto a slide. The cells were lysed, then subject to electrophoresis at 20 V for 30 min in TBE buffer. Following electrophoresis, slides were washed, dehydrated and stained with SYBR Green I. Images were taken with a fluorescent microscope and scored by CometScore software (TriTek Corporation).

### G-banding and telomere FISH of metaphase chromosome

Metaphase chromosome spreads were prepared from exponentially growing cells after treatment with demecolcine. For G-banding, the metaphase chromosomes were then treated with trypsin and stained with Giemsa according to standard procedures. Telomere FISH was performed using a Cy3-labelled peptide nucleic acid probe (Cy3-(CCCTAA)_3_) in metaphase chromosome spreads. Both the probe and the slides were heat denatured (80 °C for 5 min) and hybridized at 37 °C for 2 h. Slides were counterstained with DAPI. Images were captured using Zeiss microscope equipped with a CCD camera.

### Protein complex purification and mass spectrometry

Procedures for LRF-associated protein complex purification have been described in detail previously^51^. Briefly, FLAG-HA tandem tagged human LRF was stably expressed in HeLa cells, then nuclear extracts were sequentially immunoprecipitated with anti-FLAG and anti-HA beads. The LRF binding proteins were separated using SDS–polyacrylamide gel electrophoresis (SDS–PAGE), and protein bands were identified by mass spectrometry.

### Immunoblotting and immunoprecipitation

Cells were lysed in buffer (50 mM Tris, pH8.0, 150 mM NaCl and 0.5% NP-40). Protein concentrations of the lysates were measured by Bradford assay. The lysates were then resolved by SDS–PAGE and immunoblotted with the indicated antibodies. For immunoprecipitation, 1 mg of cell lysate was incubated with the appropriate antibodies for 3–4 h at 4 °C followed by 1-h incubation with protein A beads (Santa Cruz). Immuno-complexes were washed with buffer (20 mM Tris, pH8.0, 100 mM NaCl, 1 mM EDTA and 0.5% NP-40) before being resolved by SDS–PAGE and immunoblotted with the indicated antibodies. Uncropped scans of the most important blots are supplied as Supplementary Information.

### Cell fractionation

*p53*^*−/−*^ *Lrf*^*+/+*^ and *p53*^*−/−*^ *Lrf*^*−/−*^ cells were incubated with 100 μM phleomycin for 1 hour at 37 °C. The cell pellet was resuspended in buffer (150 mM NaCl, 50 mM Hepes PH7.5, 1 mM EDTA, 0.1% Triton X-100, protease and phosphatase inhibitor) for 10 min on ice. Lysates were pelleted, and detergent extractable supernatant collected. The pellet was further extracted with buffer (150 mM NaCl, 50 mM Hepes pH 7.5, 1 mM EDTA, 200 μg ml^−1^ RNaseA, protease and phosphatase inhibitor) for 30 min at 25 °C. Then the RNase-resistant chromatin pellet was resuspended and sonicated in buffer (150 mM NaCl, 25 mM Tris pH 7.5, 1% NP-40, 1% sodium deoxycholate, 0.1% SDS) before boiling in laemmli sampling buffer and immunoblot analysis.

### DNA repair reporter assay

2 × 10^5^ ES cells stably integrated with NHEJ or HR reporter^23^,^29^ were transfected in suspension with 0.5 μg pcDNA3β-myc NLS-I-SceI or control pcDNA3β plasmid together with 20 pmol siRNA by using Lipofectamine 2000 (Invitrogen). In the U2OS HR reporter experiments, 1 × 10^5^ cells were transiently transfected with 0.5 μg pcDNA3β-myc NLS-I-SceI or control pcDNA3β plasmid together with 20 pmol siRNA. In the U2OS NHEJ assays, 2 × 10^4^ cells were first transfected with 30 pmol siRNA in Lipofectamine RNAiMAX on day zero, then received adeno-I-SceI (MOI of 5) 48 h later (with or without the DNA-PKcs inhibitor), with readout by FACS 72 h after adeno-I-SceI transduction. Transfection efficiency was measured simultaneously by parallel transfection with wild-type GFP expression plasmid, at an amount one-tenth of the I-SceI expression vector. GFP-positive cell frequencies were measured 3 days post transfection by flow cytometry in independent replicates and corrected for transfection efficiency.

### siRNAs

Sequences of siRNAs are as follows: LRF-1: GAACCGACGACAAGGGCGU ; LRF-2: GUAUAUAGAAUGCGGAUCA ; LRF-3: CUACAGGCCUUUCGAGAUU . Brca1, Rad51, Xrcc4, hLRF are ON-TARGETplus siRNA – SMARTpool (Dharmacon). siCtrl: siGENOME Non-Targeting siRNA #2 (Dharmacon)

### Laser-induced DNA damage and fluorescence data collection

Microirradiation with a pulsed 365 nm nitrogen laser (Spectra-Physics; 365 nm, 10 Hz pulse) was used to induce DSBs in the nuclei of U2OS cells. The laser system was directly coupled (Micropoint Ablation Laser System; Photonic Instruments, Inc.) to the epifluorescence path of the microscope (Axiovert 200M (Carl Zeiss MicroImaging, Inc.) for time-lapse imaging and focused through a Plan-Apochromat × 63/NA 1.40 oil immersion objective (Carl Zeiss MicroImaging, Inc.). Laser output was set at 75% of the maximum power, equivalent to the minimal dose required to induce detectable accumulation of YFP-DNA-PKcs in living cells. Time-lapse images were acquired with an AxioCam HRm (Carl Zeiss MicroImaging, Inc.). DNA-PKcs and Ku80 kinetics were calculated as previously described^52^: fluorescence value of an undamaged spot in the same nuclei was subtracted from the fluorescence intensity of the laser-irradiated spot for every cell at each time point in order to eliminate the fluorescence background of the nucleus. Relative fluorescence intensity at each time point (RF(t)) was calculated as RF(t)=(INt−INpreIR)/(INmax−INpreIR), where IN, fluorescence intensity; INpreIR, IN of the micro-irradiated area before laser damage; INmax, maximum IN in the micro-irradiated area.

### DNA-damaging agents clonogenic survival assay

Four hundred cells were seeded in six-well plates 24 h before treatment with the indicated drugs. γ-radiation was supplied with a Cesium-137 source. After 10 days, colonies were stained with crystal violet and scored. A colony was defined as a cluster of more than ∼50 cells. Cells without drug treatment were used as control. Survival ratio=sample/control × 100%. Results were reported as mean±s.e.m. from three independent experiments.

### DNA-PK kinase assay

DNA-PK kinase activity was measured using the SignaTECT DNA-Dependent Protein Kinase Assay System (Promega). Total cell lysates were extracted using a buffer containing 1%NP-40, 150 mM NaCl and 50 mM Tris (pH8.0). Endogenous DNA of cell lysates was removed using Sepharose fast flow (GE Healthcare). For each reaction, 2 μg of cell lysates were used, reactions were incubated at 30 °C for 10 min, and then the supernatant was spotted onto a SAM membrane. DNA-PK protein kinase activity was calculated as the incorporation of ^32^P into the peptide using a phosphoimager.

### Immunofluorescence

Cells were seeded in 24-well plates containing round glass coverslips at the density of 2 × 10^4^/well. 24 h after plating, cells were fixed with paraformaldehyde, permeabilized in 0.1% Triton-X-100/phosphate buffered sulphate (PBS). Coverslips were then incubated with primary antibody diluted in 1% BSA/PBS for 1 h. After washing, coverslips were incubated in secondary antibody diluted in 1% BSA/PBS for 1 h. Coverslips were washed, stained with DAPI, mounted and analysed by confocal microscopy (Zeiss).

### Flow cytometry

Cells were trypsinised, fixed with 4% formaldehyde and permeabilised with 90% methanol. Cells were then incubated with Alexa Fluor 647 conjugated γ-H2AX antibody (Cell Signaling Technology) in 0.5% BSA /PBS for 1 h at room temperature. Cells were washed with 0.5% BSA /PBS then analysed by flow cytometry (LSR II, BD Biosciences). Data was analysed with FCS Express V3 software.

### Chromatin immunoprecipitation assay

ChIP was performed using ES cells stably integrated with NHEJ reporter. 12 h after I-SceI expression, ES cells were trypsinised, cross-linked with 1% formaldehyde at 37 °C for 10 min, and stopped in 0.125 M glycine. DNA was sonicated and incubated with indicated antibodies. Following reverse cross-linking, the associated DNA was extracted and detected by PCR using primers that locate 0.2 kb to I-SceI sites (p04.52, 5′- TGGTGAGCAAGGGCGAGGAGC -3′; p04.53, 5′- TCGTGCTGCTTCATGTGGTCG -3′) and 28 kb to I-SceI cutting sites (p07.52, 5′- TGTCATCATAGGCCCAATTTC -3′; p07.53, 5′- CCCAGTTTAAGGATGGTGGTT -3′). Using Hotstart (Qiagen) enzyme, PCR condition: 95 °C 15 min then 94 °C 30 s; 58 °C 30 s; 72 °C 30 s for 35 cycles. Samples were separated on a 2% agarose gel. ImageJ software was used to quantify the band intensity. The band intensity of PCR with IgG without I-SceI was set as 1, and the relative ratio of PCR bands (using primers locate 0.2 kb to I-SceI site)/PCR bands (using primers locate 28 kb to I-SceI site) are shown using data from three different experiments.

### RT-qPCR Primers

Total RNA was extracted with TRIzol Reagents (Invitrogen) according to the provided protocol. 1 μg total RNA was reversed transcribed with iScript cDNA Synthesis Kit (Bio-Rad). Real-time quantitative PCR was performed using diluted cDNA, SYBR Green JumpStart Taq ReadyMix (Sigma) and appropriate primers in StepOnePlus Real-Time PCR System (Applied Biosystems). Primers sequence is reported in Supplementary Table 4.

### Statistical analysis

Results are expressed as mean±s.d or s.e.m as noted. Comparisons between groups were assessed using Student's *t*-test analysis. *P*≤0.05 was considered significant.

## Additional information

**Accession codes:** The microarray data has been deposited to the Gene Expression Omnibus at the National Center for Biotechnology Information under the accession code GSE70780.

**How to cite this article:** Liu, X. S. *et al*. LRF maintains genome integrity by regulating the non-homologous end joining pathway of DNA repair. *Nat. Commun.* 6:8325 doi: 10.1038/ncomms9325 (2015).

## Supplementary Material

Supplementary Information Supplementary Figures 1-4 and Supplementary Tables 1-4
