# Supplementary material for: LRF maintains genome integrity by regulating the non-homologous end joining pathway of DNA repair
Source: Nat Commun. 2015 Oct 8;6:8325. doi: 10.1038/ncomms9325 (PMC4633636; doi:10.1038/ncomms9325)
Supplement: Supplementary Information — Supplementary Figures 1-4 and Supplementary Tables 1-4 [file ncomms9325-s1.pdf]

Supplementary Figures

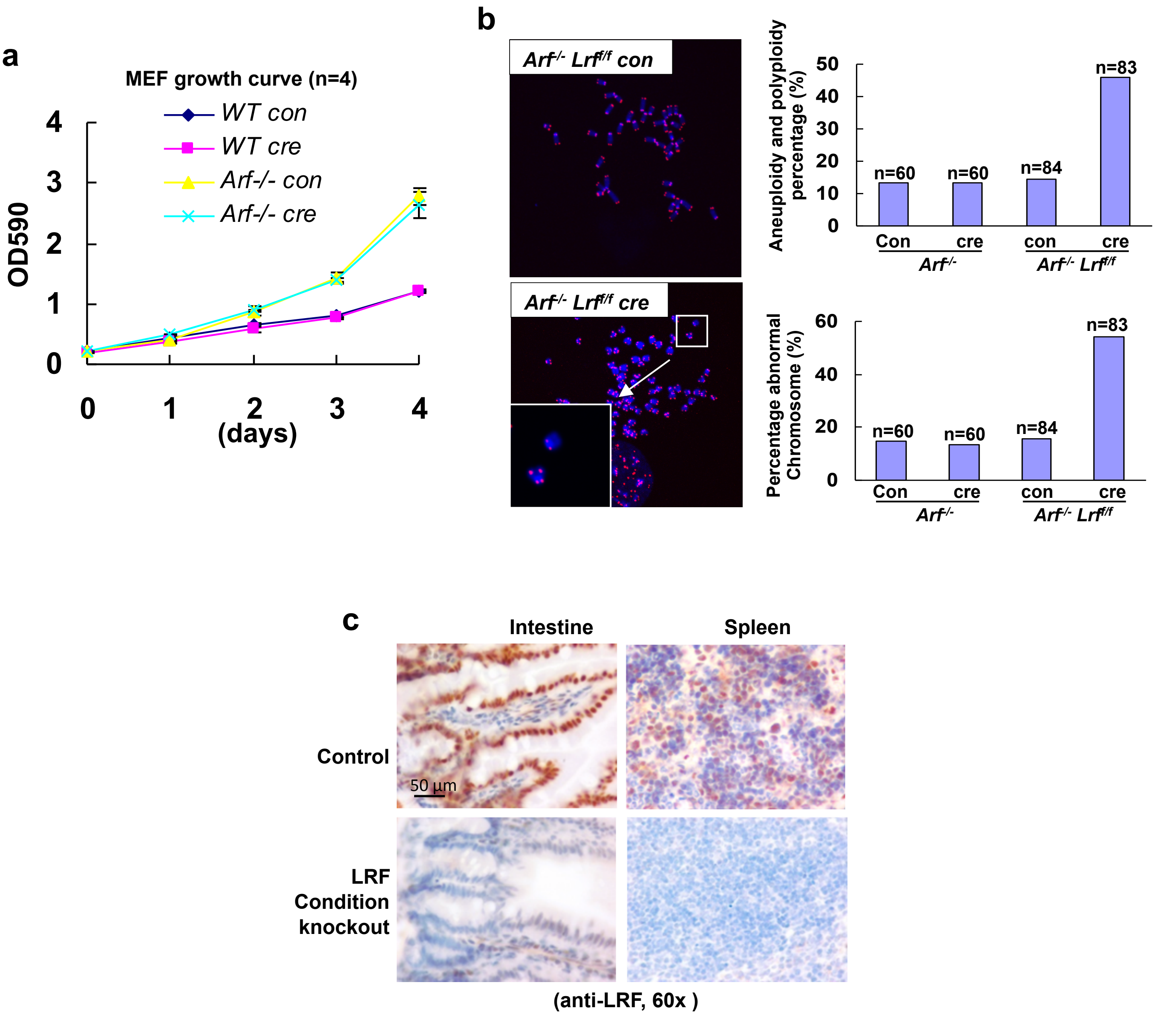

Supplementary Figure 1. LRF is required for genome integrity maintenance

(**a**) Growth curve of wild type (*WT*) and *Arf*$^{-/-}$ MEF transduced with Cre or control retrovirus. Error bars represent mean ± s.e.m. (**b**) Metaphase spreads from early passage *Arf*$^{-/-}$ and *Arf*$^{-/-}$ *Lrf* deleted MEFs were stained with telomere probes (Cy3-(CCCTAA)$_3$) and DAPI. Inserts are enlargements of a typical chromosome break. Percentages of anaeuploid and polyploid chromosomes plus abnormal chromosome structures in *Arf*$^{-/-}$ and *Arf*$^{-/-}$ *Lrf* deleted MEF are scored as indicated. n refers to the number of metaphase spreads scored. (**c**) Detection of LRF in control and *Lrf* conditional knockout intestine (*Lrf*$^{Flox/Flox}$ *Villin-Cre*, 3 months old) and spleen (*Lrf*$^{Flox/Flox}$ *Mx1-Cre*, 1 month after last pIpC) by immunohistochemistry of paraffin sections.

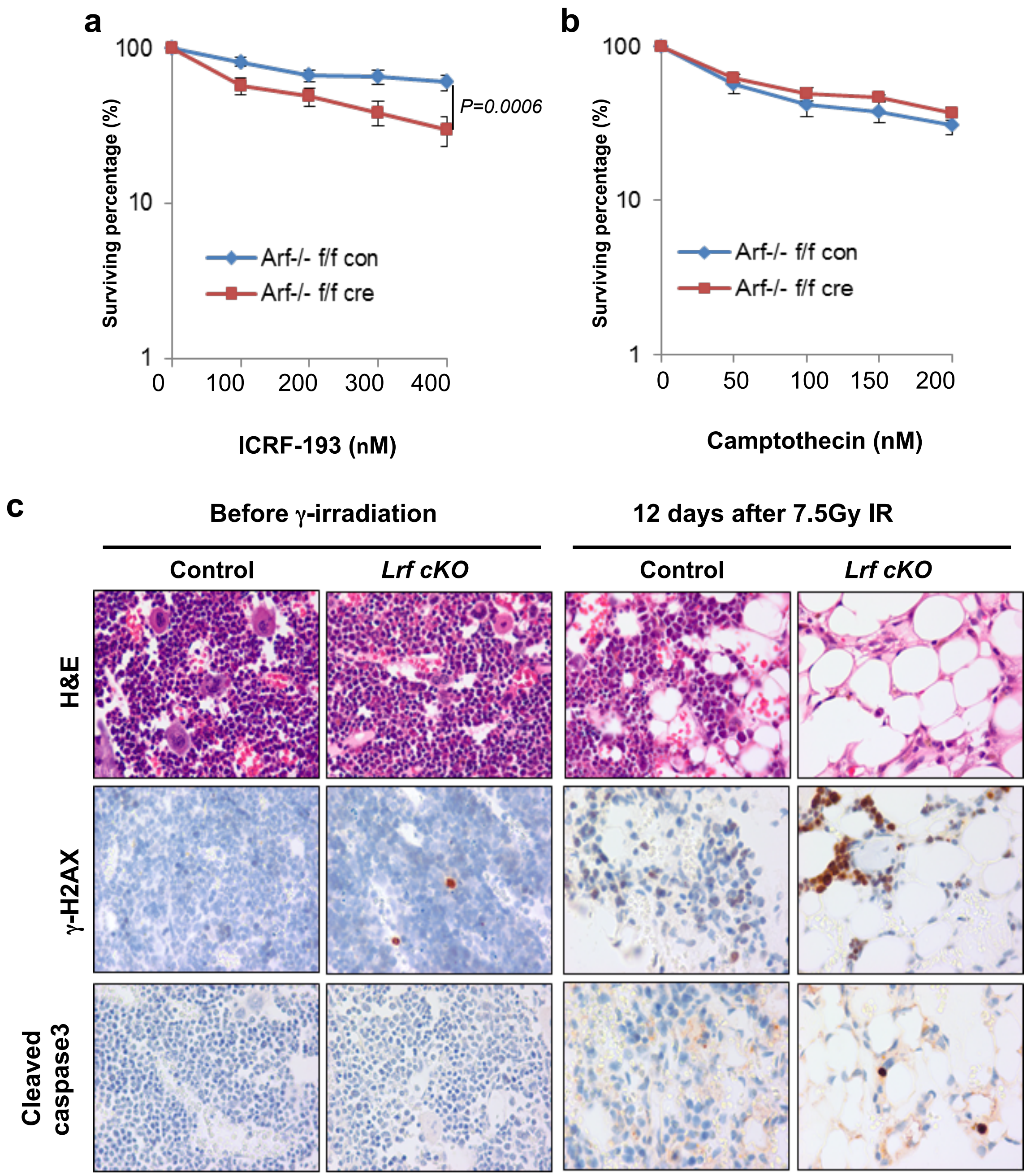

Supplementary Figure 2. LRF deficient enhances cell sensitivity to specific classes of genotoxic agents. (a-b) Clonogenic survival of Arf-/- and Arf-/- LRF deleted MEFs treated with ICRF-193 (a), and camptothecin (b). (c) Mx1-cre and pIpC induction were used to delete Lrf in mouse hematopoietic system. After pIpC induction, Mx1-Cre LrfFlox/Flox (Lrf cKO) and sibling control Mx1-Cre Lrf+/+ mice are given a single dose of whole-body g-irradiation (7.5Gy). By HE staining Lrf cKO bone marrow showed decreased cellularity compared to sibling control after IR. DNA damage level and apoptosis in bone marrow were further detected by immuno-staining with g-H2AX and cleaved caspase-3 antibodies respectively.

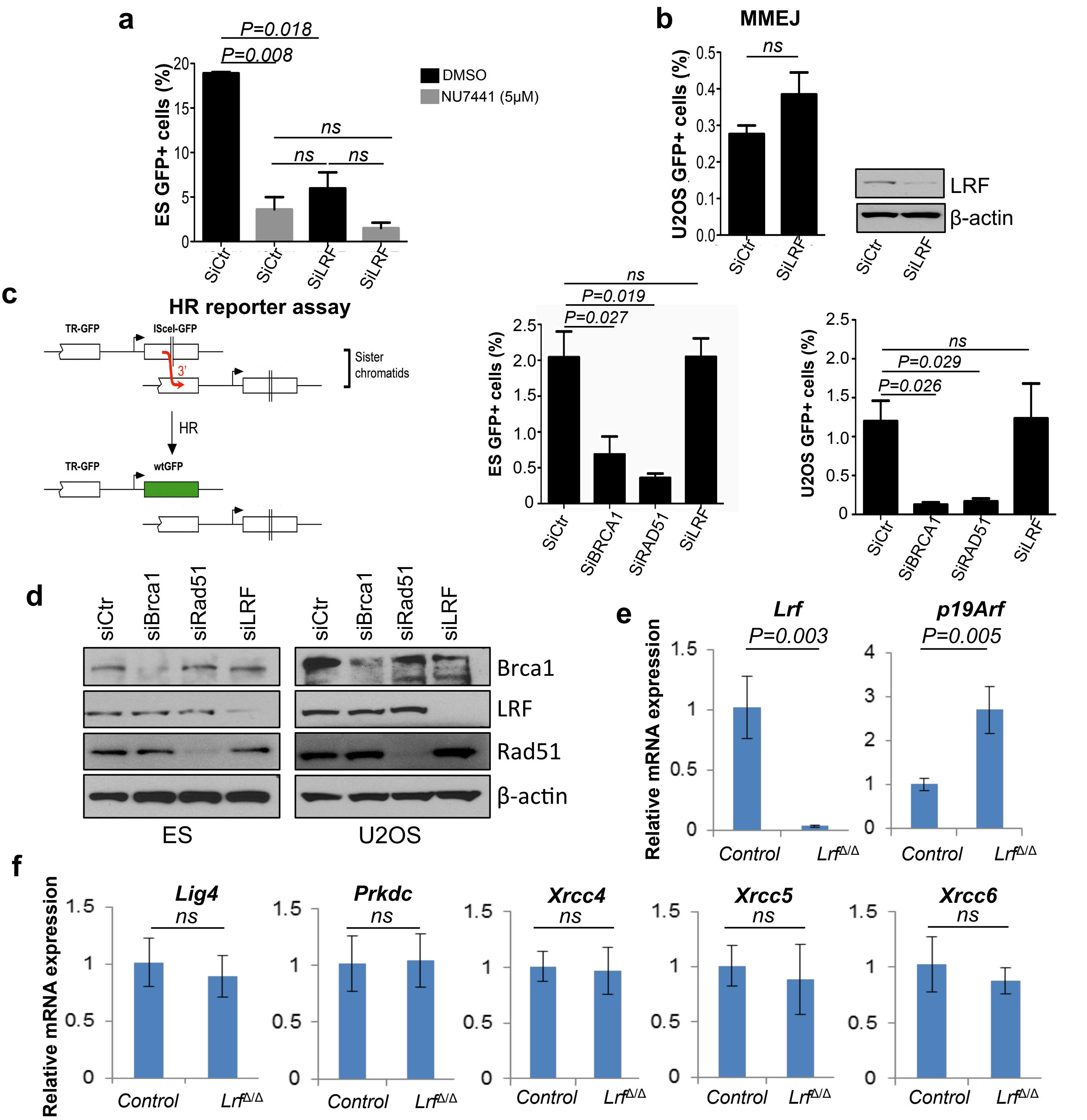

Supplementary Figure 3. Transcriptional independent function of LRF in cNHEJ. (**a**) Effect of DNA-PKcs pharmacological inhibition (NU7441) as single agent or in combination with LRF knockdown on NHEJ efficiency using a specific reporter assay (see Fig. 3a). (**b**) Effect of LRF knockdown on microhomology-mediated end joining (MMEJ) efficiency using a specific reporter assay (see Ref. #27). (**c**) Effect of LRF, Brca1, or Rad51 knockdown on homologous recombination efficiency using a specific reporter assay in ES and U2OS cells. Differences in the amount of GFP-positive cells are calculated from 4 independent experiments and presented as mean values ± s.e.m. with associated P value. (**d**) Western blot analysis showing efficiency of siRNAs used in (c). (**e-f**) RT-qPCR analysis of late passages control and *Lrf* deleted MEFs. No significant differences in mRNA levels of NHEJ genes (*Prkdc* alias Dna-PKcs; *Xrcc5* alias Ku80; *Xrcc6* alias Ku70) are detected after *Lrf* deletion. p19Arf induction is used as positive control of transcriptional derepression of Lrf targeted genes. Data are calculated from three independent experiments and presented as mean ± s.d. with associated P value.

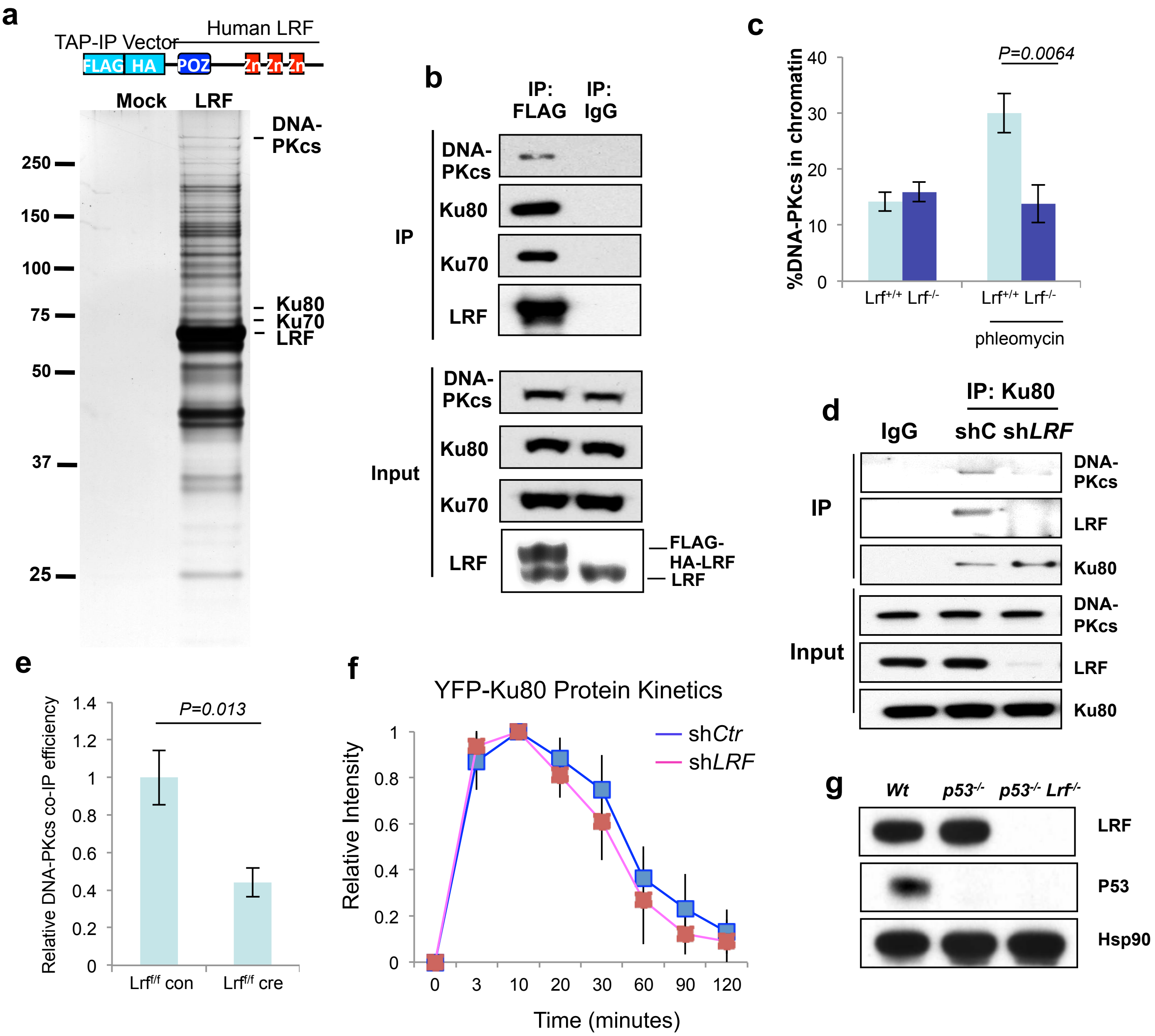

Supplementary Figure 4. Interaction of LRF with DNA-PKcs, Ku70 and Ku80

(**a**) Purification and mass spectrometric analysis of proteins associated with LRF. Nuclear extracts from HeLa cells expressing human LRF tagged with both Flag and HA epitopes were sequentially immunoprecipitated with Flag and HA antibody affinity resins. Mock-transduced HeLa cells were used as control. The LRF-associated polypeptides were detected by silver staining. The identities of associated polypeptides are indicated on the right. (**b**) FLAG antibody immunoprecipitation was performed from lysates prepared from HeLa cells stably expressing FLAG-HA-LRF. Immunoprecipitates were analysed by Western blot. (**c**) Percentage of DNA-PKcs in the chromatin fraction. Data are shown as average ± s.d of three independent experiments. (**d**) Immunoprecipitation with Ku80 antibody from shCtr or shLRF virus transduced U2OS cell lysates. Associated proteins were determined by Western blot. (**e**) Ku70/DNA-PKcs co-immunoprecipitation efficiency. Data are shown as average ± s.d of three independent experiments. (**f**) YFP tagged Ku80 was expressed in stable shCtr and shLRF U2OS cells. Association and dissociation kinetics of YFP-Ku80 recruitment to DNA damage foci are shown. Average values of 20 cells are presented as mean values ± s.d. (**g**) Western blot analysis of p53 and LRF status in wild type, p53$^{-/-}$ Lrf$^{f/f}$ ctr, and p53$^{-/-}$ Lrf$^{f/f}$ cre MEFs.

Supplementary Tables

Supplementary Table 1

ID	logFC	P.Value	Gene.Symbol
1415927_at	4.522137113	3.56E-05	Actc1 /// LOC100048431
1427004_at	3.368249387	4.35E-08	Fbxo2
1423537_at	3.225287353	1.04E-05	Gap43
1423506_a_at	2.929219414	5.96E-05	Nnat
1435275_at	2.840535173	4.64E-06	Cox6b2
1448901_at	2.797952778	4.78E-08	Cpxm1
1420940_x_at	2.60022876	9.82E-05	Rgs5
1417466_at	2.384786316	0.001913967	Rgs5
1420941_at	2.363102768	0.000800612	Rgs5
1433600_at	2.354700221	0.001007452	Adra2a
1421965_s_at	2.339895689	5.13E-06	Notch3
1428705_at	2.262426454	0.002187721	1700007K13Rik
1416357_a_at	2.213861711	0.006438428	Mcam
1418578_at	2.160906438	7.18E-05	Dgka
1419082_at	2.14545498	0.001160504	Serpinb2
1422668_at	2.095297978	4.07E-05	Serpinb9b
1416474_at	2.083297173	1.54E-05	Igdcc4
1421201_a_at	2.017909446	1.55E-07	Tro
1457825_x_at	1.972533227	0.006440087	Tcn2
1417355_at	1.971440593	0.003225144	Peg3
1417356_at	1.962140363	0.03027486	Peg3
1416006_at	1.912336837	2.54E-06	Mdk
1448154_at	1.841530066	3.67E-05	Ndrg2
1434944_at	1.826556257	0.021130965	Dmpk
1448289_at	1.806286675	0.005871462	Crmp1
1420942_s_at	1.803218762	0.002027272	Rgs5
1416271_at	1.7975339	0.007798894	Perp
1433924_at	1.764292589	2.22E-06	---
1416473_a_at	1.710087509	0.00200405	Igdcc4
1426731_at	1.701244757	0.00010808	Des
1415931_at	1.669382065	0.001030844	Igf2
1460259_s_at	1.657292742	0.004601582	Clca1 /// Clca2
1448200_at	1.640434852	0.000277694	Tcn2
1426915_at	1.61477415	0.002309169	Dapk1
1424010_at	1.607616718	0.012758705	Mfap4
1449982_at	1.605100182	2.37E-05	Il11
1442051_at	1.60118377	0.04154033	Hist2h3c1
1434325_x_at	1.599237944	0.004823109	Prkar1b
1417256_at	1.584785473	0.00801588	Mmp13
1447800_x_at	1.582255007	4.02E-05	Tcn2
1436033_at	1.546513692	0.000121673	BC031353
1424455_at	1.536149497	0.004913557	Gprasp1
1428758_at	1.531714347	0.00069771	Tmem86a
1416023_at	1.527262644	7.62E-07	Fabp3
1452249_at	1.522288791	0.000116469	Prickle1
1450784_at	1.486536877	0.011169275	Reck
1426016_a_at	1.466638963	5.34E-06	Tro
1426670_at	1.431484207	3.12E-06	Agrn
1452291_at	1.418545473	0.000484868	Arap2
1447842_x_at	1.40013298	0.000603706	Tcn2

1436197_at	1.393303855	1.48E-05	**Cdc42bpg**
1428765_at	1.386909055	0.002246027	**Meg3**
1438370_x_at	1.376469061	0.012902845	**Dos**
1435695_a_at	1.363783559	0.003456471	**Ggct**
1452284_at	1.355877561	0.047276937	**Ptprz1**
1417491_at	1.345958099	0.000151978	**Ctsb**
1450779_at	1.338200735	0.034796833	**Fabp7**
1449559_at	1.337959062	0.000708627	**Msx2**
1435943_at	1.32650517	0.003080478	**Dpep1**
1425832_a_at	1.323682174	0.000226493	**Cxcr6**
1417951_at	1.320787997	0.002556508	**Eno3**
1415996_at	1.320733763	0.040642467	**Txnip**
1421840_at	1.309539198	0.009638323	**Abca1**
1441971_at	1.305227426	0.000303054	**---**
1417649_at	1.283683582	0.00081502	**Cdkn1c**
1432417_a_at	1.273859327	0.000161909	**Tspan2**
1423854_a_at	1.273464575	0.001605986	**Rasl11b**
1417703_at	1.269742546	0.001297676	**Pvrl2**
1435595_at	1.268127031	0.028979462	**1810011O10Rik**
1418203_at	1.255482457	0.000387028	**Pmaip1**
1419613_at	1.254775928	0.014121127	**Col7a1**
1416754_at	1.246666023	0.000345551	**Prkar1b**
1422155_at	1.244857985	0.034272314	**Hist2h3c2**
1450626_at	1.237480425	0.002174226	**Manba**
1424567_at	1.22919671	0.006575294	**Tspan2**
1452106_at	1.217813631	0.002454244	**Npnt**
1416286_at	1.215126535	0.001224587	**Rgs4**
1417109_at	1.21276823	0.000133389	**Tinagl1**
1452107_s_at	1.204648567	0.010191311	**Npnt**
1440132_s_at	1.194114793	0.003902192	**Prkar1b**
1415897_a_at	1.192760517	0.007006013	**Mgst1**
1450079_at	1.186200729	0.021391659	**Nrk**
1424595_at	1.183033448	0.000147476	**F11r**
1451754_a_at	1.17763482	0.002327149	**Wdr45**
1416753_at	1.175431759	0.000479316	**Prkar1b**
1452840_at	1.173078955	0.002054295	**1500009L16Rik**
1452114_s_at	1.170221718	0.005929591	**Igfbp5**
1458268_s_at	1.15549395	0.032290267	**Igfbp3**
1423362_at	1.14956405	0.000981295	**Sort1**
1437161_x_at	1.144456599	0.007665597	**Rbpms**
1418318_at	1.129290346	0.005756254	**Rnf128**
1437458_x_at	1.128076447	0.023817511	**Clu /// LOC100046120**
1434415_at	1.124675998	0.006266761	**Dact3**
1437689_x_at	1.115368329	0.009511686	**Clu /// LOC100046120**
1450693_at	1.113142783	0.001727657	**Rgs17**
1437494_at	1.11185346	0.002713233	**Mapkapk3**
1429009_at	1.094172087	0.020219645	**Snrnp70**
1425505_at	1.084464379	0.04383733	**Mylk**
1424568_at	1.079447415	0.001823352	**Tspan2**
1416287_at	1.078407105	0.041357584	**Rgs4**
1450928_at	1.073830723	0.017461227	**LOC100045546**
1439368_a_at	1.071118986	0.046644241	**Slc9a3r2**

1426649_at	1.070241949	0.000105752	**Tmeff1**
1449965_at	1.066546558	0.000223935	**Mcpt8**
1451415_at	1.054689506	0.004189219	**1810011O10Rik**
1419456_at	1.051625927	0.014109688	**Dcxr**
1424692_at	1.047882555	0.008815235	**2810055F11Rik**
1420512_at	1.046149813	0.003638822	**Dkk2**
1452899_at	1.043443148	0.02345144	**Rian**
1436999_at	1.0350455	0.035042157	**5033414K04Rik**
1455143_at	1.032613227	9.59E-05	**Nlgn2**
1418367_x_at	1.030480326	0.012522703	**Hist1h2ad /// Hist1h2an /// Hist**
1426238_at	1.030070286	0.002794377	**Bmp1**
1423605_a_at	1.0212924	0.013140846	**Mdm2**
1424786_s_at	1.016821176	0.003590238	**Wdr45**
1419283_s_at	1.005066063	0.018370802	**Tns1**
1434815_a_at	0.999265366	9.27E-05	**Mapkapk3**
1423489_at	0.994952859	0.017089567	**LOC100047565 /// Mmd**
1421227_at	0.994365015	0.029514781	**Gzme**
1460292_a_at	0.984929107	0.006928486	**Smarca1**
1416926_at	0.980989187	0.000821862	**Trp53inp1**
1417111_at	0.9768192	0.048131165	**Man1a**
1423488_at	0.971247159	0.026505893	**Mmd**
1422033_a_at	0.968443192	0.00096503	**Cntf /// Zfp91 /// Zfp91-cntf**
1433699_at	0.963377361	0.0451559	**Tnfaip3**
1423259_at	0.962736374	0.021447135	**Id4 /// LOC100045546**
1425964_x_at	0.956403422	0.002524141	**Hspb1**
1434768_at	0.955272243	0.007865224	**Tpp1**
1421997_s_at	0.951704848	0.003297977	**Itga3**
1417979_at	0.95055337	0.008613981	**Tnmd**
1424940_s_at	0.946115708	0.00426042	**BC022687**
1422943_a_at	0.945810463	0.000739315	**Hspb1**
1452183_a_at	0.945462707	0.032212523	**Meg3**
1439925_at	0.945182654	0.045018229	**---**
1428650_at	0.943291113	0.005321115	**Tns1**
1416872_at	0.942999664	0.000467985	**Tspan6**
1417492_at	0.938228474	0.00087288	**Ctsb**
1449036_at	0.937189345	0.01434053	**Rnf128**
1449826_a_at	0.936604976	0.028681693	**Fgf2**
1455324_at	0.932917212	0.009166851	**Plcxd2**
1422476_at	0.919037985	0.003645581	**Ifi30**
1453008_at	0.917253294	0.04843931	**Trnp1**
1423309_at	0.913567327	0.013089566	**Tgoln1**
1424262_at	0.911463902	0.003659465	**Aif1l**
1416842_at	0.908387052	0.005608319	**Gstm5**
1447624_s_at	0.906171644	0.003776436	**Stox2**
1435866_s_at	0.89923794	0.012369801	**Hist3h2a**
1435745_at	0.89692552	0.000724269	**5031439G07Rik**
1416313_at	0.890874237	0.000526612	**Mllt11**
1417110_at	0.888091638	0.015415818	**Man1a**
1448169_at	0.881066105	0.00022815	**Krt18**
1449341_a_at	0.879419881	0.012814857	**Stom**
1451132_at	0.879348512	0.004690013	**LOC100046056 /// Pbxip1**
1452060_a_at	0.875416509	0.008114763	**Limk2**

1424111_at	0.875199262	0.006888323	**Igf2r**
1448830_at	0.873857454	0.004289405	**Dusp1**
1432591_at	0.873376075	0.010395835	**Pappa**
1420994_at	0.869272723	0.014693861	**B3gnt5**
1419112_at	0.864549505	0.001325209	**Nlk**
1428065_at	0.860615956	0.000570643	**Slc44a2**
1417311_at	0.860430165	0.032850566	**Crip2**
1451731_at	0.859821053	0.038921665	**Abca3**
1419458_at	0.856777343	0.040443588	**Rgnef**
1448919_at	0.853471001	0.013687794	**Cd302**
1435970_at	0.85187852	0.002444254	**Nlk**
1455768_at	0.849012459	0.007819327	**Npc2**
1436297_a_at	0.848145695	0.00181939	**Grina**
1425536_at	0.847187526	0.000308206	**Stx3**
1440085_at	0.844264697	0.008659507	**Eda2r**
1428902_at	0.839684424	0.013262695	**Chst11**
1456479_at	0.838350255	0.047372723	**Snx30**
1448501_at	0.837708527	0.00088564	**Tspan6**
1423278_at	0.832444592	0.034107998	**Ptprk**
1447711_x_at	0.830874032	0.017689532	**4933412E12Rik**
1418412_at	0.827766773	0.004747789	**Tpd52l1**
1456005_a_at	0.826666779	0.005456526	**Bcl2l11**
1421594_a_at	0.825776207	0.019296732	**Sytl2**
1422340_a_at	0.822308893	0.042806986	**Actg2**
1415911_at	0.82186488	0.001022618	**Impact**
1440522_at	0.821791905	0.012183967	**ENSMUSG00000073019**
1455166_at	0.819740745	0.016322211	**Arl5b**
1416326_at	0.814973745	6.72E-05	**Crip1**
1448316_at	0.813835263	0.004074127	**Cmtm3 /// LOC100046883**
1452796_at	0.80824118	0.045331684	**Def6**
1427069_at	0.806742901	0.002082901	**Fbxo28**
1423306_at	0.804957574	0.000132014	**2010002N04Rik**
1434059_at	0.803272982	0.010972308	**B230312A22Rik**
1424114_s_at	0.80231732	0.000264095	**Lamb1-1**
1433545_s_at	0.80161576	0.001991819	**Acad11 /// Nphp3**
1435083_at	0.795840737	0.002429406	**Ctxn1**
1435945_a_at	0.794785332	0.014027782	**Kcnn4**
1435580_at	0.790709295	0.00289607	**C230081A13Rik**
1418483_a_at	0.787984812	0.001204741	**Ggta1**
1453173_at	0.787452456	0.001420637	**2310005E10Rik**
1455505_at	0.786658337	0.001631475	**Gatad2a**
1433808_at	0.784502879	0.000868418	**Heatr7a**
1449048_s_at	0.776838869	0.008425068	**Rab4a**
1436304_at	0.776730728	0.020665647	**C030003D03Rik**
1436050_x_at	0.776483415	0.007328621	**Hes6**
1424777_at	0.775349662	0.002792212	**Wdr21**
1420664_s_at	0.775103581	0.006594141	**Procr**
1418379_s_at	0.771894243	0.004275673	**Gpr124**
1460344_at	0.77080107	0.000664696	**2310033F14Rik**
1429060_at	0.76363855	0.001912143	**Malat1**
1423994_at	0.759280983	0.007904683	**Kif1b**
1433725_at	0.759197676	0.00095759	**Acvr1b**

1456393_at	0.758680793	0.008106391	**2310002J21Rik**
1434586_a_at	0.7554146	0.011123254	**Ptdss2**
1434272_at	0.754874062	0.030769982	**Cpeb2**
1419089_at	0.75140337	0.010069366	**Timp3**
1436838_x_at	0.750846653	0.011580067	**Cotl1**
1416261_at	0.749521846	0.002401684	**Tmem19**
1439380_x_at	0.745114257	0.003432884	**Meg3**
1422293_a_at	0.741279796	0.008033295	**Kctd1**
1452905_at	0.738640949	0.006382612	**Meg3**
1448468_a_at	0.738144573	0.028211234	**Kcnab1**
1416408_at	0.737182688	0.00603404	**Acox1**
1419428_a_at	0.734745034	0.014468462	**Gaa**
1434196_at	0.734127142	0.008134094	**Dnaja4**
1416262_at	0.732439164	0.014610213	**Tmem19**
1418172_at	0.731576998	0.018728171	**Hebp1**
1451756_at	0.728080771	0.042034011	**Flt1**
1425506_at	0.727548352	0.010014641	**Mylk**
1417271_a_at	0.727298851	0.002966283	**Eng**
1434657_at	0.726986502	0.037211189	**Gls**
1428110_x_at	0.725105798	0.04616079	**Vps11**
1424996_at	0.724704375	0.000244027	**Cflar**
1449405_at	0.715338576	0.042925935	**Tns1**
1448812_at	0.712810756	0.009677209	**Hpcal1**
1448428_at	0.711729972	0.004069669	**Nbl1**
1434832_at	0.711030573	0.004199919	**Foxo3**
1422607_at	0.708364576	0.02041479	**Etv1**
1422552_at	0.707004871	0.002910769	**Rprm**
1436640_x_at	0.703796661	0.000234711	**Agpat4**
1456945_at	0.700802193	0.001822542	**Nudt6**
1452251_at	0.700361849	0.048720416	**Nbea**
1415834_at	0.700271111	0.016297851	**Dusp6**
1421088_at	0.699703952	0.001286982	**Gpc4**
1460036_at	0.698423274	0.003548429	**Ap1s2**
1417237_at	0.693992193	6.46E-05	**Pld2**
1424970_at	0.69377176	0.033813613	**Purg**
1433843_at	0.692574199	0.000169336	**Hs1bp3**
1418711_at	0.692112341	0.011803878	**Pdgfa**
1419630_a_at	0.688814753	0.013847581	**Trim11**
1448529_at	0.686798915	0.013010974	**Thbd**
1423212_at	0.686673049	0.010561502	**Phc1**
1425357_a_at	0.682760404	0.041893024	**Grem1**
1424638_at	0.67987868	0.003167036	**Cdkn1a**
1423608_at	0.6789298	0.021409704	**Itm2a**
1431094_at	0.678587423	0.016687686	**1110006E14Rik**
1451381_at	0.678078322	0.001557822	**1810020D17Rik**
1417644_at	0.677769878	0.009732032	**Sspn**
1436890_at	0.677326636	0.00365704	**Uap1l1**
1439259_x_at	0.677013691	0.002220327	**Abhd4**
1451196_at	0.676555829	0.000432804	**Ypel5**
1422603_at	0.675813728	0.001181753	**Rnase4**
1451747_a_at	0.675466818	0.027651391	**Atg12**
1433773_at	0.675457692	0.021408605	**Rrm2b**

1432158_a_at	0.674673485	0.015455739	Trappc2
1439388_s_at	0.674098841	0.006205139	Bcar1
1428503_a_at	0.67325679	0.023431172	Nkiras1
1416666_at	0.670338159	0.019841278	Serpine2
1437120_at	0.660805621	0.026015821	Snx30
1434394_at	0.659469007	0.025724157	N4bp2
1436849_x_at	0.65678327	0.003224007	Gaa
1418072_at	0.656401145	0.001054556	Hist1h2bc
1426792_s_at	0.656143573	0.003268887	Rusc2
1419814_s_at	0.655494555	0.045176918	S100a1
1415977_at	0.654944525	0.004501271	Isyna1
1451554_a_at	0.654839639	0.001716281	Aph1a
1425673_at	0.654793692	0.016921396	Lpp
1424842_a_at	0.652639041	0.027288424	Arhgap24
1426481_at	0.650133549	0.000635062	Klhl22
1444232_at	0.649924388	0.017063264	Prkg1
1434487_at	0.648255962	0.002607833	Mef2d
1434831_a_at	0.648224956	0.001311022	Foxo3
1448313_at	0.647761039	0.02116451	Tpp1
1448272_at	0.647430756	0.002947596	Btg2
1460556_at	0.646525653	0.013891351	Micall1
1418402_at	0.644201836	0.024596473	Adam19 /// LOC100045780
1434162_at	0.644179486	0.003459052	2700078E11Rik
1452059_at	0.643995096	0.008145444	Slc35f5
1423995_at	0.638547964	0.008656445	Kif1b
1423071_x_at	0.636753787	0.000298948	6720475J19Rik
1416588_at	0.636398296	0.017007087	Ptprn
1460346_at	0.635566339	0.003137958	Arsa
1437345_a_at	0.631455255	0.026805603	Bscl2
1448163_at	0.630840137	0.003022726	Gnpda1
1436514_at	0.629411238	0.013799288	Gpc4
1452847_at	0.629251622	0.015773878	Gatsl3
1426516_a_at	0.629001649	0.042998434	Lpin1
1416686_at	0.627801618	0.00367927	Plod2
1434587_x_at	0.6238591	0.000834612	Ptdss2
1419971_s_at	0.623771831	0.024919308	Slc35a5
1416488_at	0.623592544	0.011076531	Ccng2
1425632_a_at	0.621751004	0.001664317	Pqlc2
1448477_at	0.619666193	0.016166319	Chst12
1450935_at	0.619518495	0.003432335	Ercc5
1449324_at	0.617362178	0.011415713	Ero1l
1426241_a_at	0.617228998	0.012061769	Scmh1
1417599_at	0.614837629	0.020467611	Cd276
1424126_at	0.613888372	0.019980909	Alas1
1433737_at	0.611794894	0.005944316	LOC677213 /// Uhmk1
1433757_a_at	0.611106996	0.01618206	Nisch
1427573_at	0.610706585	0.029744199	Chic1
1417850_at	0.610020729	0.044628999	Rb1
1434126_at	0.607424461	0.009835824	---
1415961_at	0.606982612	0.007725756	Itm2c
1422168_a_at	0.605771867	0.004566634	Bdnf
1426728_x_at	0.605487575	0.0118426	Ptdss2

1448525_a_at	0.604320781	0.017273365	**Bnip3l**
1433751_at	0.60381396	0.015227028	**Slc39a10**
1455002_at	0.601591538	0.01559996	**Ptp4a1**
1416340_a_at	0.601170928	0.023215933	**Man2b1**
1424246_a_at	0.600851193	0.03077455	**Tes**
1448946_at	0.600617347	0.009713532	**Kif3c**
1428120_at	0.600530893	0.011035358	**Fbxw9**
1457465_at	0.599576942	0.038022785	**Shroom4**
1451525_at	0.598008121	0.003811757	**Arhgap12**
1437341_x_at	0.597682185	0.011692722	**Cnp**
1426615_s_at	0.595830207	0.021668434	**Ndrg4**
1438910_a_at	0.595620408	0.018741644	**Stom**
1422692_at	0.595099945	0.041571715	**Sub1**
1416344_at	0.592551833	0.004572546	**Lamp2**
1448857_a_at	0.58864995	0.002315748	**Zdhhc12**
1424726_at	0.587673335	0.016602937	**Tmem150**
1426857_a_at	0.586995301	0.014933819	**Hsdl2**
1426708_at	0.586470462	0.002492568	**Antxr2**
1448655_at	0.585847507	0.014763912	**Lrp1**
1454792_s_at	-0.587571114	0.003958113	**Sephs1**
1433960_at	-0.588736255	0.024229927	**Isg20l2**
1459651_s_at	-0.591694749	0.021985622	**Nup54**
1450034_at	-0.591783717	0.048883277	**Stat1**
1447623_s_at	-0.59196103	0.049230862	**Prkd1**
1432394_a_at	-0.591997111	0.004268349	**Aatf**
1416073_a_at	-0.592787968	0.018802344	**Nup85**
1416152_a_at	-0.595911923	0.003375322	**Sfrs3**
1424136_a_at	-0.596347604	0.04589572	**LOC433064 /// Ppih**
1456251_x_at	-0.597120191	0.020709241	**Tspo**
1456236_s_at	-0.597581434	0.006107359	**Commd10**
1428264_at	-0.60314544	0.038781918	**Snrnp40**
1453745_at	-0.604010206	0.029075468	**2700038G22Rik**
1437378_x_at	-0.605859774	0.041828794	**Scarb1**
1460687_at	-0.608434869	0.001278827	**Setd8**
1415673_at	-0.610346135	0.0342757	**Psph**
1416415_a_at	-0.610722457	0.02743809	**H2afz**
1415964_at	-0.610908748	0.010496076	**Scd1**
1452598_at	-0.611988584	0.002129954	**Gins1**
1455084_x_at	-0.613105611	0.015473564	**Shmt2**
1434241_at	-0.613458899	0.003269351	**Wdr67**
1417621_at	-0.614505105	0.014916267	**Nfatc1**
1418737_at	-0.615167921	0.000495384	**Nudt2**
1434305_at	-0.615234547	0.004204448	**Tmem149 /// U2af1l4**
1425531_at	-0.616140012	0.025937078	**Znhit1**
1438320_s_at	-0.616378369	0.001494808	**Mcm7**
1449348_at	-0.617268885	0.025220928	**Mpp6**
1452785_at	-0.617817826	0.006179035	**1700034H14Rik**
1427680_a_at	-0.618615682	0.018835912	**Nfib**
1424318_at	-0.62440187	0.014930912	**1110067D22Rik**
1438678_at	-0.62518722	0.022188474	**1500011K16Rik**
1421142_s_at	-0.627877042	0.027556094	**Foxp1**
1419076_a_at	-0.627980158	0.041545829	**Brca2**

1448938_at	-0.628227356	0.00653144	Rpa3
1425048_a_at	-0.630305131	0.001664522	Hmgb1
1433856_at	-0.631573354	0.041523266	Hisppd1
1426609_at	-0.634989711	0.003206096	Dis3
1436802_at	-0.635498191	0.015324879	Ilf3
1423289_a_at	-0.635949139	0.016570651	1810029B16Rik
1428656_at	-0.639928032	0.028400725	LOC100045148 /// Rnasen
1423577_at	-0.64094978	0.021327183	Ankrd32
1424201_a_at	-0.641062855	0.012085226	Seh1l
1419690_at	-0.64251403	0.001488385	2610002M06Rik
1424542_at	-0.643593885	0.001484754	S100a4
1436161_at	-0.644316575	0.044874363	Pds5b
1427364_a_at	-0.644449446	0.028858184	Odc1
1429624_at	-0.646313476	0.029978045	Sltm
1428452_at	-0.646982446	0.015912717	2810025M15Rik
1422503_s_at	-0.647162861	0.005224612	Parp1
1455083_at	-0.647967346	0.012599866	Atp11c
1436842_at	-0.6483318	0.038013194	B230380D07Rik
1422628_at	-0.651755479	0.002492125	4632417K18Rik
1460403_at	-0.656553507	0.008909922	Psip1
1452062_at	-0.65887456	0.039511375	Prpsap2
1417404_at	-0.660295151	0.000183227	Elovl6
1424556_at	-0.662566898	0.010879871	Pycr1
1423524_at	-0.662955256	0.011076623	Mastl
1434239_at	-0.663075091	0.011188055	Rrp12
1423625_a_at	-0.664095849	0.006261875	Dnajc19
1455007_s_at	-0.664848374	0.046779334	Gpt2
1438360_x_at	-0.66509595	0.045417791	EG383528 /// EG433326 /// Slc2
1456032_x_at	-0.666106218	0.035537186	EG666634 /// H2afz
1420592_a_at	-0.667798349	0.000560632	Anp32e
1417457_at	-0.67061825	0.004109018	Cks2
1423969_at	-0.670620242	0.003009664	Nup37
1418380_at	-0.670816842	0.009250005	Terf1
1428440_at	-0.671177482	0.013677703	Slc25a12
1460353_at	-0.673773861	0.001257725	Tmem48
1455887_at	-0.674819651	0.02782685	Alg8
1430134_a_at	-0.67572505	0.039827819	Yars2
1450522_a_at	-0.67794783	0.046922309	H1f0
1452590_a_at	-0.678546242	0.01872378	ENSMUSG00000043248 /// Plac9
1428114_at	-0.67889688	0.03326547	Slc14a1
1423702_at	-0.678922978	0.013390709	H1f0
1417458_s_at	-0.683756886	0.000419425	Cks2
1427020_at	-0.685579409	0.008090561	Scara3
1450694_at	-0.686610461	0.016769195	Fkbp2
1428582_at	-0.688416534	0.010011417	Mettl10
1425615_a_at	-0.691795796	0.023153472	Pck2
1428213_at	-0.692268937	0.000172656	Nsmce4a
1438096_a_at	-0.693024731	0.010722995	Dtymk
1448635_at	-0.69314902	0.002662148	Smc2
1449705_x_at	-0.694286888	0.007583242	LOC100045677 /// Mcm3
1422430_at	-0.696168546	0.031393431	Fignl1
1416251_at	-0.698427975	0.041842417	Mcm6

1434699_at	-0.698731314	0.02880914	G2e3
1417373_a_at	-0.70062851	0.00906554	Tuba4a
1424895_at	-0.702393638	0.016048249	Gpsm2
1433935_at	-0.702542088	0.048806755	AU020206
1423809_at	-0.707296483	0.013359245	Tcf19
1429028_at	-0.708073337	0.016013476	Dock11
1437829_s_at	-0.70968219	0.004878367	Eef2k
1423138_at	-0.709747984	0.004729611	Wdr4
1419130_at	-0.715139902	0.000561708	Adat2
1428410_at	-0.715771545	0.015618679	Nat13
1428061_at	-0.715903628	0.003784169	Hat1
1426363_x_at	-0.719208517	0.015840753	H2afy2
1438092_x_at	-0.71999017	0.028289431	H2afz
1450943_at	-0.720307955	0.008756343	Magohb
1418152_at	-0.720883218	0.040195525	Nsbp1
1423371_at	-0.721500213	0.034036144	Pole4
1434555_at	-0.726995756	0.034859549	Anp32a
1452036_a_at	-0.727358554	0.001368921	Tmpo
1425179_at	-0.72820919	0.036260522	Shmt1
1448314_at	-0.729830929	0.000550398	Cdc2a
1424107_at	-0.733316473	0.030389926	Kif18a
1416176_at	-0.733415908	7.34E-05	Hmgb1
1421731_a_at	-0.737916443	0.026602446	Fen1
1422510_at	-0.740500924	0.005066031	Ctdspl
1428593_at	-0.740830241	0.004218914	1700029F09Rik
1418281_at	-0.742361133	0.00210102	Rad51
1429712_at	-0.743393583	0.04623273	Etohi1
1454877_at	-0.746480536	0.003391607	Sertad4
1450959_at	-0.748642384	0.022709108	D930014E17Rik
1423525_at	-0.750233502	0.044038569	Mastl
1428679_s_at	-0.750356125	0.032574683	0610010K14Rik
1436349_at	-0.75327645	0.000507111	2700094K13Rik
1416166_a_at	-0.754039897	0.019848161	Prdx4
1416031_s_at	-0.755696218	0.001300306	Mcm7
1415849_s_at	-0.756565654	0.00016879	Stmn1
1434422_at	-0.762341149	0.024991638	1700066M21Rik
1439269_x_at	-0.763766812	0.035386535	Mcm7
1437716_x_at	-0.764075955	0.00041514	Kif22
1428450_at	-0.764170267	0.040284028	2610034B18Rik
1435926_at	-0.769232973	0.047526879	Chml
1451602_at	-0.772375139	0.031339294	Snx6
1425761_a_at	-0.772441088	0.00214265	Nfatc1
1435114_at	-0.775500917	0.015214575	Wdhd1
1439377_x_at	-0.776869862	0.001599444	Cdc20
1424643_at	-0.779170698	0.001265143	Tcof1
1438434_at	-0.781096884	0.003537868	Arhgap11a
1434695_at	-0.783600674	0.024873329	Dtl
1415772_at	-0.786817199	0.002079442	Ncl
1448288_at	-0.787922325	0.022065196	Nfib
1421861_at	-0.789078525	0.000214805	Clstn1
1438091_a_at	-0.789488552	0.022584704	EG626950 /// H2afz
1448638_at	-0.790149807	0.041599738	Mtbp

1417822_at	-0.792404775	0.009685647	D17H6S56E-5
1422533_at	-0.794083181	0.004117686	Cyp51
1416748_a_at	-0.795325275	0.000759885	Mre11a
1433543_at	-0.796620283	0.000309788	Anln
1429491_s_at	-0.796775921	0.015604354	Rif1
1434602_at	-0.799544375	0.046762531	Med13l
1436248_at	-0.80172358	0.007245675	Rasal2
1424991_s_at	-0.80258189	0.003097248	Tyms /// Tyms-ps
1425815_a_at	-0.807044593	0.044749313	Hmmr
1417541_at	-0.810515715	0.045636116	Hells
1447704_s_at	-0.812658493	0.038897066	Utp23
1455488_at	-0.813071594	0.018495404	Haus6
1418334_at	-0.815103978	0.000655193	Dbf4
1416125_at	-0.81909382	0.019674859	Fkbp5
1425006_a_at	-0.82070325	0.000693529	Vrk1
1429364_at	-0.820873552	0.023886938	4930579G24Rik
1415829_at	-0.822069516	0.000460646	Lbr
1428672_at	-0.826163395	0.003076378	Snrpf
1423055_at	-0.827416922	0.000891589	Nsg1
1434462_at	-0.828943498	0.001544548	BC088983
1417719_at	-0.829570106	0.003693769	Sap30
1418326_at	-0.829754817	0.013537325	LOC100047619 /// Slc7a5
1428386_at	-0.831148159	0.012484452	Acsl3
1426002_a_at	-0.832336245	0.010457004	Cdc7
1459546_s_at	-0.832399535	0.019517527	Enpp1
1417403_at	-0.832411053	0.005603902	Elovl6
1423680_at	-0.837431492	0.005266854	Fads1
1427541_x_at	-0.839176466	0.013301466	Hmmr
1417374_at	-0.840766123	0.000407392	Tuba4a
1420028_s_at	-0.843229214	0.038718386	LOC100045677 /// Mcm3
1456471_x_at	-0.843698509	0.011600745	EG385344 /// EG627427 /// EG6
1422535_at	-0.845144051	0.042436875	Ccne2
1417506_at	-0.85007848	0.003294397	Gmnn
1422016_a_at	-0.850628215	0.002496027	Cenph
1415878_at	-0.857982414	0.000732806	Rrm1
1454671_at	-0.86263714	0.024613553	Insig1
1433813_at	-0.863040242	0.000601409	---
1433482_a_at	-0.865650658	0.000165534	Fubp1
1415802_at	-0.867203031	0.00021285	Slc16a1
1450241_a_at	-0.870948074	0.041102783	Evi2a
1447640_s_at	-0.871750269	0.005360479	Pbx3
1460603_at	-0.874244802	0.046585165	Samd9l
1424511_at	-0.877307712	0.027790319	Aurka
1451103_at	-0.877308144	0.041752301	Haus4
1421918_at	-0.878050447	0.03379842	Anp32a
1455841_s_at	-0.885921443	0.013007337	Grwd1
1439394_x_at	-0.886594227	4.09E-05	Cdc20
1417166_at	-0.889070261	0.000748316	Psip1
1429295_s_at	-0.889429471	0.000752748	Trip13
1425050_at	-0.891485673	0.007216069	Isoc1
1451080_at	-0.893859386	0.000514229	Usp1
1455011_at	-0.894069132	0.009540986	Stard4

1416641_at	-0.895744587	0.00103251	**Lig1**
1436174_at	-0.89661158	0.007887067	**Atad2**
1455730_at	-0.90442522	0.000208744	**Dlgap5**
1423920_at	-0.905607854	0.00261736	**Ncaph**
1423774_a_at	-0.906035954	0.044197218	**Prc1**
1419153_at	-0.908828263	0.040921373	**2810417H13Rik**
1418264_at	-0.910290352	0.03800568	**Cenpk**
1428104_at	-0.912043869	0.001236186	**Tpx2**
1416575_at	-0.912780513	0.002625417	**Cdc45l**
1423620_at	-0.915993602	0.00044626	**Cenpq**
1452305_s_at	-0.916302636	0.004593805	**Cenpn**
1427094_at	-0.922280483	0.006630429	**Pole2**
1439852_at	-0.927417325	0.047707418	**---**
1418026_at	-0.928205016	0.026249272	**Exo1**
1426937_at	-0.930023241	0.010896254	**6330406I15Rik**
1425053_at	-0.933891377	0.000543982	**Isoc1**
1419513_a_at	-0.934098284	0.001258935	**Ect2**
1450112_a_at	-0.934130984	0.012296922	**Gas2**
1456055_x_at	-0.93492687	0.009589124	**Pold1**
1422317_a_at	-0.935316599	0.026244733	**Il1rl1**
1439442_x_at	-0.935691114	0.049664398	**Yars2**
1435938_at	-0.936374426	0.004274594	**Ckap2l**
1439436_x_at	-0.93811243	0.005818786	**Incenp**
1424766_at	-0.938196495	0.00839611	**Ercc6l**
1438016_at	-0.941227054	0.012813107	**Dkc1**
1452771_s_at	-0.947177425	0.022271566	**Acsl3**
1427275_at	-0.951657262	0.00066008	**Smc4**
1452242_at	-0.953720897	0.000783707	**Cep55**
1424321_at	-0.954113184	0.001284897	**Rfc4**
1456653_a_at	-0.955543959	0.012838777	**Mthfd1l**
1416354_at	-0.961644573	0.000826268	**Rbmx**
1424971_at	-0.966856822	0.000167131	**Ccdc99**
1438161_s_at	-0.966972692	0.011492222	**Rfc4**
1417938_at	-0.971222772	0.005817241	**Rad51ap1**
1417019_a_at	-0.971369745	0.024924724	**Cdc6**
1427105_at	-0.971662891	0.001262768	**Cenpn**
1423775_s_at	-0.973008466	0.000419471	**Prc1**
1452197_at	-0.973086773	8.55E-05	**Smc4**
1426349_s_at	-0.975675912	0.014577932	**Tmpo**
1424278_a_at	-0.976491936	0.000185173	**Birc5**
1453007_at	-0.977296446	0.041224371	**3110082I17Rik**
1422460_at	-0.979909718	0.000640376	**Mad2l1**
1429172_a_at	-0.98511408	0.008363303	**Ncapg**
1434102_at	-0.98720589	0.047473643	**Nfib**
1434850_at	-0.989888176	0.002888393	**Iqgap3**
1437370_at	-0.990183177	0.033893817	**Sgol2**
1425052_at	-0.991613242	4.89E-05	**---**
1428105_at	-0.994423364	0.003730057	**Tpx2**
1423847_at	-0.999328508	0.000147088	**Ncapd2**
1452534_a_at	-1.00139025	0.039236116	**Hmgb2**
1419838_s_at	-1.002315593	0.012133163	**Plk4**
1433408_a_at	-1.003908368	0.00766046	**Mcm10**

1416309_at	-1.011299033	0.000701594	Nusap1
1428069_at	-1.012620841	0.022890011	Cdca7
1416299_at	-1.01354471	0.007405705	Shcbp1
1452458_s_at	-1.018264489	0.036708481	Ppil5
1419397_at	-1.021043136	0.00096096	Pola1
1454694_a_at	-1.02370488	0.001572669	Top2a
1429499_at	-1.025531646	0.017874415	Fbxo5
1417926_at	-1.02839111	0.00479001	Ncapg2
1453107_s_at	-1.032132579	0.018870596	4933413G19Rik /// Foxm1 /// P
1417586_at	-1.035149204	0.007388181	Timeless
1436847_s_at	-1.036093689	0.003390292	Cdca8
1448650_a_at	-1.041360854	0.001067294	Pole
1441788_s_at	-1.042646306	0.002114276	Dkc1
1418369_at	-1.046584222	0.00018142	Prim1
1428976_at	-1.047559207	0.000925721	Tmpo
1434767_at	-1.050259537	0.015747114	C79407
1421952_at	-1.051167208	0.026844241	Capn6
1416802_a_at	-1.052322671	0.000895435	Cdca5
1442058_s_at	-1.056462708	0.021959026	Psmc3ip
1455990_at	-1.069112503	0.000129187	Kif23
1418337_at	-1.075672247	0.018355353	Rpia
1421612_a_at	-1.085884007	0.000625515	H2afy2 /// H2afy3
1418919_at	-1.088380084	0.002662179	Sgol1
1448205_at	-1.091178554	4.95E-05	Ccnb1
1419152_at	-1.093403475	0.002336887	2810417H13Rik
1449556_at	-1.094275956	0.000448891	C920025E04Rik /// H2-T23
1439040_at	-1.100427501	0.029559586	Cenpe
1460713_at	-1.100947356	4.66E-05	BC048355
1428706_at	-1.111472689	0.000141864	Cenpv
1452115_a_at	-1.112233123	0.002505323	Plk4
1449061_a_at	-1.113992877	0.014465193	Prim1
1419270_a_at	-1.115966306	3.74E-05	Dut
1460041_at	-1.116525421	0.002063521	Flrt1
1448899_s_at	-1.118373869	0.004771266	Rad51ap1
1437313_x_at	-1.120791354	0.002569043	Hmgb2
1449207_a_at	-1.123211578	0.002295358	Kif20a
1434678_at	-1.126080756	0.003431044	Mbnl3
1422944_a_at	-1.128914261	0.009767301	Diap3
1448277_at	-1.131987122	0.013755047	Pold2
1450156_a_at	-1.140871369	0.018942218	Hmmr
1415965_at	-1.142483701	0.00070044	Scd1
1426580_at	-1.142998333	0.000355457	Plk4
1429294_at	-1.146761098	0.000387267	Trip13
1426817_at	-1.14780132	0.000435468	Mki67
1449171_at	-1.148575813	4.65E-05	Ttk
1417910_at	-1.154179132	0.0005355	Ccna2
1417911_at	-1.156758388	2.39E-05	Ccna2
1437251_at	-1.158450939	0.032468177	Cdca2
1433893_s_at	-1.160311871	0.001325363	Spag5
1448191_at	-1.160677005	0.047078106	Plk1
1427161_at	-1.16600077	0.002509524	Cenpf
1417445_at	-1.168160393	3.90E-05	Ndc80

1420913_at	-1.174442939	0.028435329	**Slco2a1**
1417450_a_at	-1.175231044	1.32E-05	**Tacc3**
1416076_at	-1.176625131	0.022912016	**Ccnb1 /// EG434175 /// EG6670(**
1425923_at	-1.176773824	9.21E-05	**Mycn**
1430811_a_at	-1.180012302	2.38E-05	**Nuf2**
1422814_at	-1.183139294	0.015233796	**Aspm**
1456280_at	-1.188047154	0.01315685	**Clspn**
1416558_at	-1.188659396	0.000135918	**Melk**
1437611_x_at	-1.190955479	0.000666787	**Kif2c**
1450692_at	-1.191679296	2.81E-05	**Kif4**
1451358_a_at	-1.199359903	0.003950019	**Racgap1**
1428481_s_at	-1.204365264	0.032071544	**Cdca8**
1435005_at	-1.212979594	0.00666217	**Cenpe**
1450842_a_at	-1.215600601	0.000254018	**Cenpa**
1424810_at	-1.215830358	0.006172716	**Tasp1**
1424046_at	-1.21955484	6.75E-05	**Bub1**
1450496_a_at	-1.224951843	0.000498884	**2810433K01Rik**
1450920_at	-1.226471857	5.88E-06	**Ccnb2**
1436808_x_at	-1.227613381	0.002488012	**Mcm5**
1453769_at	-1.227755158	0.038918716	**Ckap2l**
1437580_s_at	-1.2323175	0.002037488	**Nek2**
1419943_s_at	-1.235114428	0.000383863	**Ccnb1**
1416868_at	-1.237775529	0.001614484	**Cdkn2c**
1429171_a_at	-1.243638395	0.000553196	**Ncapg**
1416258_at	-1.248021358	0.011289356	**Tk1**
1421546_a_at	-1.253146401	0.013909818	**Racgap1**
1449699_s_at	-1.25471068	0.000960591	**C330027C09Rik**
1439695_a_at	-1.269277436	0.0045351	**Kif20b**
1458374_at	-1.278154294	0.001360841	**C79407**
1423813_at	-1.285003415	0.032771457	**Kif22**
1416155_at	-1.313054901	0.000408558	**Hmgb3**
1429156_at	-1.313876517	1.84E-05	**2610036L11Rik**
AFFX-r2-Bs-thr-3_s_at	-1.314109051	0.034645053	**---**
1451128_s_at	-1.317627148	0.024817951	**Kif22**
1420081_s_at	-1.32944179	0.001127724	**D2Ertd750e**
1416961_at	-1.3326798	0.004543869	**Bub1b**
1452040_a_at	-1.35286786	1.97E-05	**Cdca3**
1424128_x_at	-1.362467979	0.004947869	**Aurkb**
1434278_at	-1.394078745	5.62E-05	**Mtm1**
1436119_at	-1.402242373	0.007707682	**Aldh1l2**
1434279_at	-1.402924198	0.001198249	**---**
1452314_at	-1.446405441	0.005149536	**Kif11**
1452315_at	-1.459827153	0.047608296	**Kif11**
1434936_at	-1.462746514	0.00340202	**Hirip3**
1454904_at	-1.509559885	0.00966211	**Mtm1**
1416715_at	-1.514775187	7.11E-06	**Gjb3**
1423463_a_at	-1.517152703	0.010650123	**D2Ertd750e**
1447363_s_at	-1.534634719	1.23E-05	**Bub1b**
1423607_at	-1.560952442	0.00309225	**Lum**
1428480_at	-1.587264037	0.043215266	**Cdca8**
1423271_at	-1.589179423	0.002346466	**Gjb2**
1430574_at	-1.638887318	0.000180655	**Cdkn3**

1456077_x_at	-1.642850386	7.83E-05	**Cdc25c**
1431087_at	-1.671740876	7.21E-05	**Spc24**
1448627_s_at	-1.672969833	1.14E-05	**Pbk**
1424156_at	-1.759224987	0.000189853	**Rbl1**
1434280_at	-1.864863582	0.000102323	**---**
1437347_at	-2.19872036	0.00457744	**Ednrb**
AFFX-r2-Bs-dap-3_at	-2.451112122	0.03897167	**---**

Supplementary Table 2

Gene name	Protein name	LogFC	P value
Cdkn2a	Arf	0.465248945	0.069607788
c-NHEJ related genes			
Prkdc	DNA-PKcs	0.295641403	0.190634798
Xrcc6	KU70	-0.075229039	0.740943812
Xrcc5	KU80	-0.207424684	0.270165102
Lig4	DNA ligase IV	-0.045251619	0.461391908
Xrcc4	XRCC4	-0.358699515	0.248707726
alt-NHEJ related genes			
Lig3	DNA ligase III	-0.124671111	0.229675825
Xrcc1	XRCC1	-0.358699515	0.248707726
Mre11a	Mre11	-0.795325275	0.000759885
Rad50	Rad50	-0.187257786	0.26646666
Nbn	Nbs1	-0.562804488	0.06507075

Supplementary Table 3

Protein name	Protein symbol
Actin-like protein 6A	ACTL6A
AT-rich interactive domain-containing protein 1A	ARID1A
T-complex protein 1 subunit delta	CCT4
T-complex protein 1 subunit zeta	CCT6A
Chromodomain-helicase-DNA-binding protein 4	CHD4
Chromodomain-helicase-DNA-binding protein 8	CHD8
DNA damage-binding protein 1	DDB1
Nucleolar RNA helicase 2	DDX21
Probable ATP-dependent RNA helicase DDX31	DDX31
ATP-dependent RNA helicase DDX39	DDX39
ATP-dependent RNA helicase A	DHX9
Probable dimethyladenosine transferase	DIMT1L
DNA-dependent protein kinase catalytic subunit	DNA-PKcs
Dynamin-1-like protein	DNM1L
Dynamin-2	DNM2
Histone-lysine N-methyltransferase, H3 lysine-9 specifi	EHMT2
Eukaryotic initiation factor 4A-I	EIF4A1
Eukaryotic initiation factor 4A-III	EIF4A3
Fatty acid synthase	FASN
Transcriptional repressor p66-alpha	GATAD2A
Translational activator GCN1	GCN1L1
General transcription factor II-I	GTF2I
General transcription factor 3C polypeptide 1	GTF3C1
Trifunctional enzyme subunit alpha, mitochondrial	HADHA
Trifunctional enzyme subunit beta, mitochondrial	HADHB
Histone H2B type 1-J	HIST1H2BJ
Heterogeneous nuclear ribonucleoproteins A2/B1	HNRNPA2B1
Heterogeneous nuclear ribonucleoprotein H	HNRNPH1
Heterogeneous nuclear ribonucleoprotein M	HNRNPM
Heterogeneous nuclear ribonucleoprotein U	HNRNPU
Heat shock protein HSP 90-alpha	HSP90AA1
Heat shock 70 kDa protein 1	HSPA1A
Heat shock cognate 71 kDa protein	HSPA8
E3 ubiquitin-protein ligase HUWE1	HUWE1
Ras GTPase-activating-like protein IQGAP1	IQGAP1
XRCC6	KU70
XRCC5	KU80
Lamin-A/C	LMNA
Melanoma-associated antigen D2	MAGED2
Midasin	MDN1
MAX gene-associated protein	MGA
Metastasis-associated protein MTA2	MTA2
Myb-binding protein 1A	MYBBP1A
Poly [ADP-ribose] polymerase 1	PARP1
6-phosphofructokinase, muscle type	PFKM
6-phosphofructokinase type C	PFKP
Histone-binding protein RBBP7	RBBP7
RNA-binding protein 39	RBM39
Replication factor C subunit 1	RFC1
Replication factor C subunit 4	RFC4

Replication protein A 70 kDa DNA-binding subunit	RPA1
60S ribosomal protein L27	RPL27
60S ribosomal protein L28	RPL28
60S ribosomal protein L35a	RPL35A
60S ribosomal protein L7	RPL7
40S ribosomal protein S14	RPS14
40S ribosomal protein S17	RPS17
40S ribosomal protein S19	RPS19
40S ribosomal protein S8	RPS8
RuvB-like 1	RUVBL1
RuvB-like 2	RUVBL2
Splicing factor 3B subunit 1	SF3B1
SHC-transforming protein 1	SHC1
Structural maintenance of chromosomes protein 3	SMARCA4
SWI/SNF-related matrix-associated actin-dependent re	SMARCA5
SWI/SNF complex subunit SMARCC2	SMARCC2
Structural maintenance of chromosomes protein 1A	SMC1A
Structural maintenance of chromosomes protein 2	SMC2
Structural maintenance of chromosomes protein 3	SMC3
Structural maintenance of chromosomes protein 4	SMC4
Lamina-associated polypeptide 2, isoform alpha	TMPO
E3 ubiquitin-protein ligase UBR5	UBR5
Vimentin	VIM
Protein Wiz	WIZ
Zinc finger and BTB domain-containing protein 4	ZBTB4
Zinc finger and BTB domain-containing protein 43	ZBTB43
Zinc finger and BTB domain-containing protein 7B	ZBTB7B/ThPok

Supplemenatry Table 4

p19Arf-F	CGGAATCCTGGACCAGGTG
p19Arf-R	ACCAGCGTGTCCAGGAAGC
Prkdc-F	AAACCTGTTCCGAGCTTTTCTG
Prkdc-R	TCTCAATCTGAGGACGAATTGC
Xrcc4-F	CTTGCTTCTGAACCCAACGTA
Xrcc4-R	TGGCCGTCAGTAAGTGTAATAAC
Xrcc5-F	ATGGCGTGGTCCGGTAATAAG
Xrcc5-R	CCTGTCGTTGGACAAACATAGTC
Xrcc6-F	ATGTCAGAGTGGGAGTCCTAC
Xrcc6-R	TCGCTGCTTATGATCTTACTGGT
Lig4-F	ATGGCTTCCTCACAAACTTCAC
Lig4-R	TTTCTGCACGGTCTTTACCTTT
Lrf-F	ATCTGCGAGAAGGTGATTCAGG
Lrf-R	ATGTGCACCTTCAGCTTGTC
18s-F	AGGGGAGAGCGGGTAAGAGA
18s-R	GGACAGGACTAGGCGGAACA
